# Sodium Fluoride Exposure Leads to ATP Depletion and Altered RNA Decay in Escherichia coli under Anaerobic Conditions

**DOI:** 10.1128/spectrum.04158-22

**Published:** 2023-03-20

**Authors:** Oleg N. Murashko, Kun-Hai Yeh, Chen-Hsin Albert Yu, Vladimir R. Kaberdin, Sue Lin-Chao

**Affiliations:** a Institute of Molecular Biology, Academia Sinica, Taipei, Taiwan; b Department of Immunology, Microbiology and Parasitology, University of the Basque Country UPV/EHU, Leioa, Spain; c Basque Foundation for Science, IKERBASQUE, Bilbao, Spain; d Research Centre for Experimental Marine Biology and Biotechnology (PIE-UPV/EHU), Plentzia, Spain; University of Manitoba

**Keywords:** sodium fluoride, gene regulation, mRNA degradation, posttranscriptional regulation, anaerobic growth

## Abstract

Although fluoride-containing compounds are widely used to inhibit bacterial growth, the reprogramming of gene expression underlying cellular responses to fluoride, especially under anaerobic conditions, is still poorly understood. Here, we compare the genome-wide transcriptomic profiles of E. coli grown in the absence (control) or presence (20 and 70 mM) of sodium fluoride (NaF) under anaerobic conditions and assess the impact of fluoride-dependent ATP depletion on RNA turnover. Tiling array analysis revealed transcripts displaying altered abundance in response to NaF treatments. Quantile-based K-means clustering uncovered a subset of genes that were highly upregulated and then downregulated in response to increased and subsequently decreased fluoride concentrations, many of which (~40%) contained repetitive extragenic palindromic (REP) sequences. Northern blot analysis of some of these highly upregulated REP-containing transcripts (i.e., *osmC*, *proP*, *efeO* and *yghA*) confirmed their considerably enhanced abundance in response to NaF treatment. An mRNA stability analysis of *osmC* and *yghA* transcripts demonstrated that fluoride treatment slows down RNA degradation, thereby enhancing RNA stability and steady-state mRNA levels. Moreover, we demonstrate that turnover of these transcripts depends on RNase E activity and RNA degradosome. Thus, we show that NaF exerts significant effects at the whole-transcriptome level under hypoxic growth (i.e., mimicking the host environment), and fluoride can impact gene expression posttranscriptionally by slowing down ATP-dependent degradation of structured RNAs.

**IMPORTANCE** Gram-negative Escherichia coli is a rod-shaped facultative anaerobic bacterium commonly found in microaerobic/anaerobic environments, including the dental plaques of warm-blooded organisms. These latter can be treated efficiently with fluoride-rich compounds that act as anticaries agents to prevent tooth decay. Although fluoride inhibits microbial growth by affecting metabolic pathways, the molecular mechanisms underlying its activity under anaerobic conditions remain poorly defined. Here, using genome-wide transcriptomics, we explore the impact of fluoride treatments on E. coli gene expression under anaerobic conditions. We reveal key gene clusters associated with cellular responses to fluoride and define its ATP-dependent stabilizing effects on transcripts containing repetitive extragenic palindromic sequences. We demonstrate the mechanisms controlling the RNA stability of these REP-containing mRNAs. Thus, fluoride can affect gene expression posttranscriptionally by stabilizing structured RNAs.

## INTRODUCTION

Escherichia coli is a Gram-negative, facultative anaerobic, rod-shaped bacterium that can be found in diverse natural environments, including water, sediment, and mud. However, the most common habitat of E. coli is in the gastrointestinal tract of humans and other warm-blooded organisms, though its presence has also been reported in dental plaques and inflammatory tissues (1–3). These animal habitats are predominantly microaerobic/anaerobic, necessitating that E. coli deploys an alternative metabolic mode to those living in other natural environments. E. coli is exposed to the action of various molecules/chemicals in these varied and dynamic environments, including fluoride-containing compounds, which are used extensively as anticaries agents. The antimicrobial action of fluoride ions is best known for its ability to inhibit bacterial growth and key metabolic pathways, including glycolysis (4). This metabolic pathway is especially important under anaerobic conditions when glucose is used as the sole carbon source. Moreover, glycolysis plays the main role in energy production under anaerobic conditions, i.e., in the absence of respiration. Glycolysis is a central, evolutionarily conserved metabolic pathway that generates ATP by converting glucose to pyruvate under both aerobic and anaerobic conditions (5). This metabolic pathway is particularly essential under the anaerobic conditions encountered by E. coli and other pathogenic bacteria in their animal hosts. It has long been known that fluoride ions inhibit the activities both the glycolytic enzyme enolase (6) and gluconeogenetic enzyme phosphoenolpyruvate synthetase (7). Enolase is a key glycolytic enzyme involved in converting 2-glycerol phosphate to phosphoenolpyruvate (5). Owing to its significant role in metabolism, enolase is essential for sustaining bacterial growth. E. coli mutants possessing a disrupted *eno* gene cannot grow without additional media supplements, such as pyruvate, glycerate or succinate. Moreover, ATP synthesis is diminished upon NaF treatment (8), which impairs or even completely inhibits cell growth. The impact of NaF on E. coli is bacteriostatic, so reducing NaF concentration in the environment can reverse the effect (9, 10).

E. coli and other enterobacterial pathogens can encounter much higher concentrations of fluoride (e.g., due to exposure to fluoride compounds present in toothpaste) in the oral cavity than in the intestine. We chose E. coli as representative of the enterobacteria, that along with many other species, are components of the human oral microbiota. Presence of E. coli in the oral cavity and its association with several diseases has been reported previously (11–13).

In general, fluoride-mediated inhibition of glycolysis and subsequently diminished ATP production can potentially affect many ATP-dependent mechanisms, such as those involved in nucleic acid and protein biogenesis as well as RNA turnover. In E. coli, RNA degradation is controlled by an RNase E-based protein complex, the RNA degradosome, whose major components are RNase E, enolase, RhlB helicase, and PNPase (14–17). Presence of RNA degradosomal assemblies is required for normal RNA turnover and cell growth under both aerobic (18) and anaerobic (19) conditions. Moreover, degradosome-associated RhlB helicase possesses ATP-dependent activity for unwinding structured RNAs, thereby facilitating their further degradation by PNPase (17). Although stable structures in RNAs can act as a barrier to the RNA degradation machinery, details of this phenomenon under anaerobic conditions and in ATP-depleted bacteria remain obscure.

Apart from interfering with ATP production to impair bacterial growth, the antimicrobial action of fluoride ions can also be related to their inhibition of other enzymes, including F-ATPases, sulfatases, catalases, and phosphatases, among others (20). Nevertheless, the degree to which NaF-dependent inhibition of these enzymes affects bacterial physiology has not yet been addressed comprehensively. Although fluoride ions are generally toxic to bacteria, the mechanisms that can mitigate the negative effects of this ion are primarily studied under aerobic conditions, and very little is known about cellular responses under anaerobic conditions.

Given all of these considerations, we investigated the global effect of fluoride ions on anaerobic E. coli by comparing the transcriptome profiles of untreated and NaF-treated cells, allowing us to explore the impact of NaF-dependent ATP depletion on RNA decay.

## RESULTS

### Sodium fluoride treatment of anaerobically grown E. coli leads to ATP depletion.

**FIG 1:**
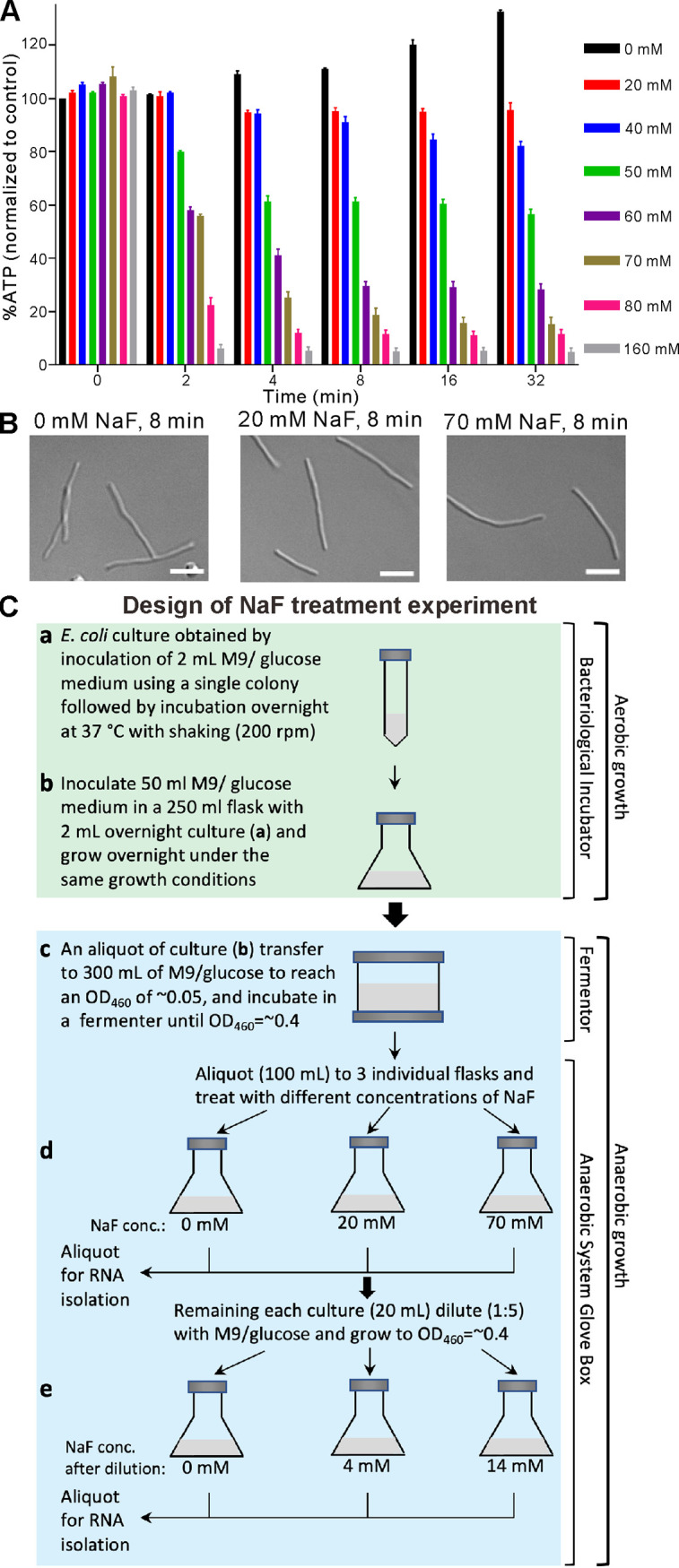
ATP levels and cell morphology in E. coli in the presence or absence of NaF under anaerobic conditions and gene expression analysis workflow. (A) ATP levels in E. coli grown under anaerobic conditions in response to NaF treatment (0 [control] to 160 mM). Mean values (from three biological replicates) expressed as percentages of ATP in each sample (normalized to the concentration of ATP in the control culture [0 mM, 0 min]). Error bars represent standard deviation. (B) Effect of NaF on cell morphology. E. coli cells were incubated in the absence (control, 0 mM) or presence of 20 or 70 mM NaF for 8 min under anaerobic conditions and analyzed by confocal microscopy. Size bars correspond to 5 μm. (C) Workflow of sample preparation for tiling array. Two sets of cultures (Cc, Cd and Cd, Ce, respectively) were grown under anaerobic conditions using M9/glucose medium.

It has long been known that fluoride ions can interrupt glycolysis by inhibiting the activities of the glycolytic enzyme, enolase (6) and the gluconeogenetic enzyme, phosphoenolpyruvate synthetase (7). Experiments on eukaryotic cells have shown that inhibition of these enzymes reduces ATP levels (8). Here, we deployed increasing NaF concentrations (0, 20, 40, 50, 60, 70, 80, and 160 mM) to study ATP depletion in E. coli under anaerobic conditions. We cultured the E. coli MG1655 strain on minimal medium (M9) containing 0.4% glucose in a 1 L Winpact Bench-Top fermentor, as described previously (19), and then measured ATP levels in the cultures. We found that ATP levels gradually declined in response to increasing NaF concentration and prolonged incubation (Fig. 1A). In particular, we found that treatment with 80 or 160 mM NaF led to a sharp decrease (~5- or 10-fold, respectively) in ATP level even after only 2 min, which remained low for up to 32 min of treatment. In contrast, at NaF concentrations in the range of 20 to 70 mM, ATP levels declined more gradually, reaching a minimal level after ~8 min of treatment and remaining low thereafter. To study the impact of NaF treatment, we chose to compare the effects of 20 mM NaF and 70 mM NaF after 8-min treatments (causing moderate and profound ATP depletion, respectively) versus control (0 mM NaF) in further experiments. Moreover, to determine if NaF treatment is accompanied by any phenotypic change, we also examined cell morphology, which revealed that untreated (control) and NaF-treated cells displayed a similar filamentous morphology (i.e., cell length >5 μm; Fig. 1B), mimicking the cell morphology of anaerobically grown E. coli MG1655 (19). Under the aforementioned experimental conditions, there were no obvious differences in cell size, appearance or integrity, indicating that our NaF treatments did not elicit any morphological changes in cells intended for further study of the impact of NaF on gene expression in E. coli grown anaerobically.

### Preparation of anaerobic cultures and genome-wide gene expression patterns under different NaF treatments.

To analyze the impact of NaF by tiling array, we prepared two sets of cultures in M9/glucose medium. The first one (Cd cultures) included the untreated cell culture (0 mM NaF, control), as well as two cultures treated with either 20 or 70 mM NaF (Fig. 1C). The second set of cultures (Ce cultures) focused on partly reversing the effects of NaF treatment by diluting the Cd cultures with fresh M9/glucose medium and further incubating them to reach the cell density of the original (undiluted) cultures (Fig. 1C). To obtain both sets of cultures, cells were grown anaerobically, as described previously (19), with details of our culture conditions and reagents provided in Materials and Methods. Total RNA was extracted using a hot acidic phenol method (21). After checking total RNA quality, it was converted to labeled cDNA and hybridized with a whole-genome tiling array (NimbleGen) to determine gene expression. These experiments were performed on three biological replicates of each culture. Tiling array signals were mapped to the E. coli K-12 substrain MG1655 reference genome (NC_000913.3) (22), allowing us to detect the expression of 4,417 genes in NaF-treated and control E. coli cells under anaerobic conditions (NCBI GEO accession no. GSE211579).

### Differential expression of genes in NaF-treated E. coli cells under anaerobic conditions.

**FIG 2:**
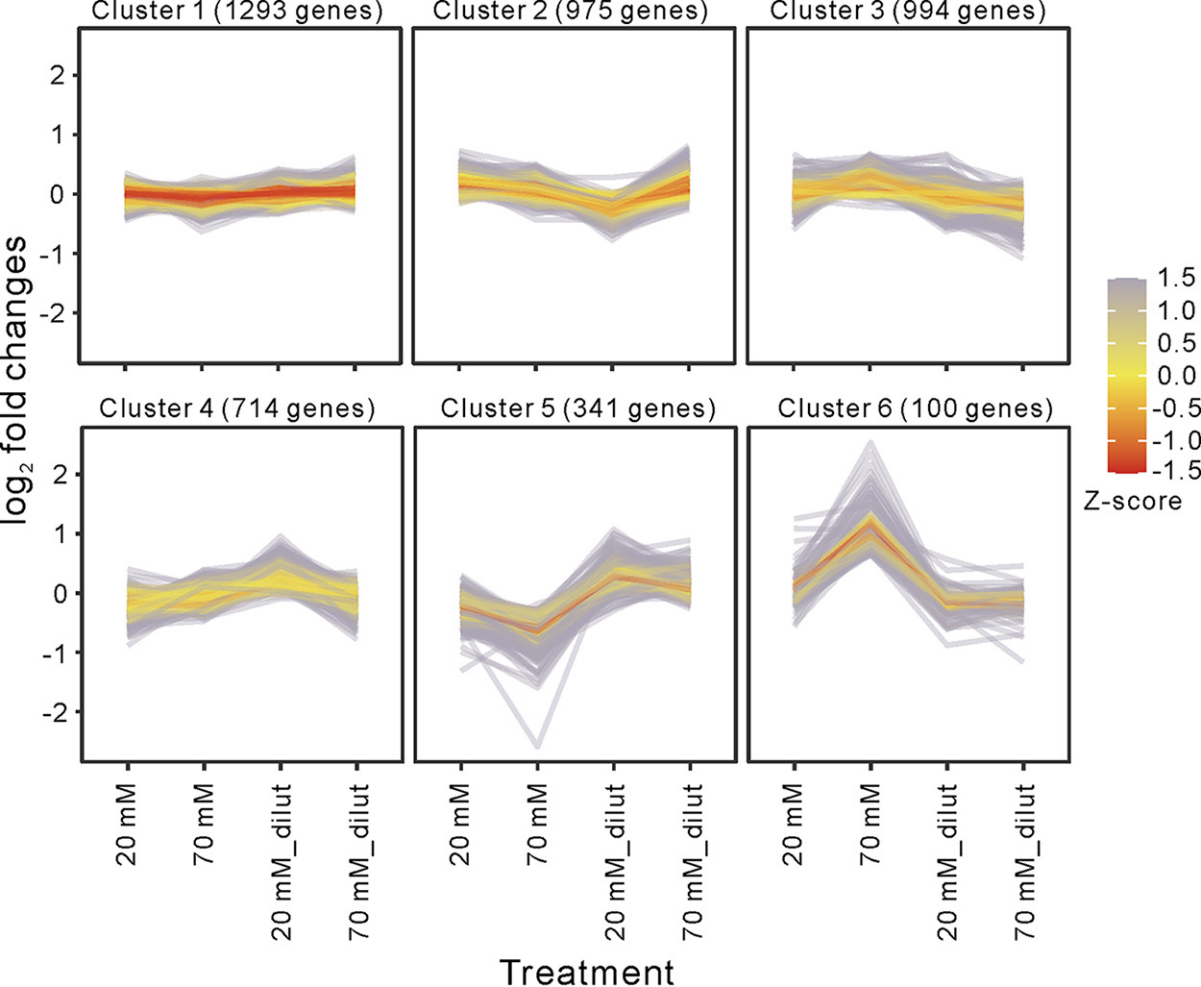
K-means clustering of gene expression data reflecting the dose-dependent effects of NaF on differential gene expression in E. coli under anaerobic conditions. Expression profiles obtained from four treatment conditions (20, 70, 20 mM_dilut [5× diluted from 20 mM] and 70 mM_dilut [5× diluted from 70 mM] mM NaF) were clustered by the K-means algorithm with k = 6. The red lines represent gene expression profiles that are well supported based on their Z-scaled log_2_ Euclidean distance to the mean of the cluster.

**FIG 3:**
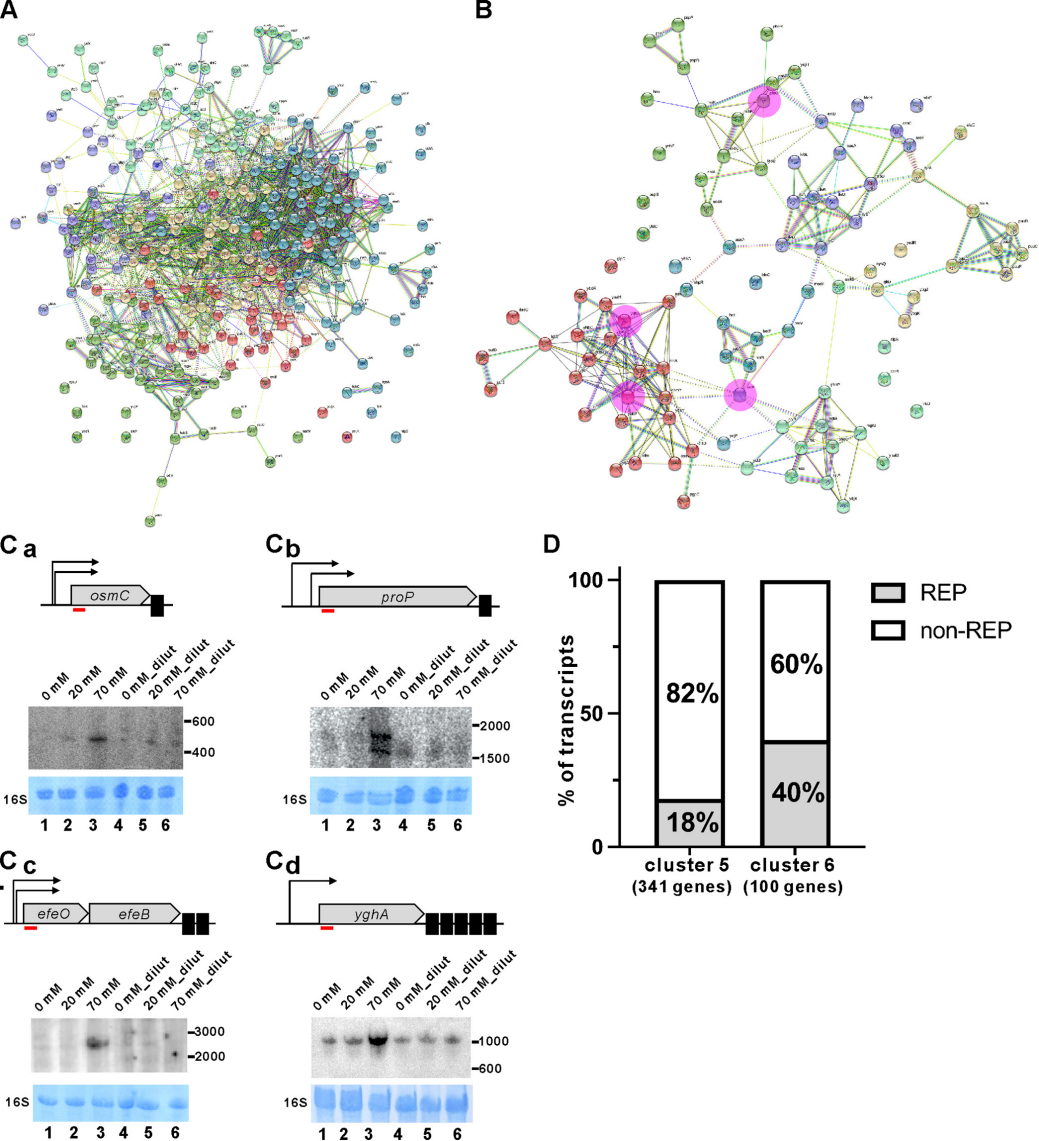
Functional association analysis of genes transiently downregulated (cluster 5) or upregulated (cluster 6) as a result of increased and subsequently decreased NaF levels. (A and B) The functional interaction networks of upregulated (A) and downregulated (B) genes of cluster 5 and 6, respectively, were generated using the functional enrichment analysis available at STRING (https://string-db.org/). Network nodes are labeled with gene symbols and nodes of the same color indicate that the corresponding proteins are involved in the same biological pathway. The lines represent functional interactions between proteins. Pink highlighted nodes in panel B indicate proteins whose cognate transcripts were selected for Northern blot validation, presented in panel C. (C) Transient accumulation of *osmC* (Ca), *proP* (Cb), *efeO* (Cc), and *yghA* (Cd) RNAs upon an increase (0, 20, and 70 mM) and subsequent decrease (0, 4 [20 mM_dilut] and 14 [70 mM_dilut]) in concentration of NaF. Hybridizations were performed with internally labeled probes specific to selected RNAs from cluster 6. The 16S rRNA served as an internal loading control. The RiboRuler RNA ladder (Thermo Scientific) was used as a size marker. Schematic representations of the *osmC*, *proP*, *efeO-efeB*, and *yghA* operons are shown on top of each Northern blot. Repetitive extragenic palindromic sequences (REPs) are depicted as black rectangles. The regions complementary to the probes are indicated by thin red lines under the 5′ end of each transcript. (D) Relative percentages of REP-containing RNAs versus RNAs lacking REPs in clusters 5 and 6.

**(i) Clustering of differentially expressed genes.** We used a quantile-based K-means clustering approach to identify gene clusters with similar expression profiles arising from NaF treatment (Fig. 2). That analysis of the expression data revealed six clusters displaying distinct profiles, of which cluster 1 (*n* = 1,293 genes), cluster 2 (*n* = 975), cluster 3 (*n* = 974), and cluster 4 (*n* = 714) did not show any differences (log_2_ fold changes) in gene expression potentially correlated with the level of NaF in the examined cultures. In contrast, gene expression notably decreased in cluster 5 (*n* = 341) and increased in cluster 6 (*n* = 100) in a dose-dependent manner in response to NaF treatment (Fig. 2). We used the functional gene categories assigned according to the STRING platform (https://string-db.org/) to assess the significance of enriched expression of the functionally related genes in clusters 5 and 6 (Fig. 3A and B). Intriguingly, we found that twice as many transcripts in cluster 6 carried repetitive extragenic palindromic (REP) sequences than those in cluster 5 (Fig. 3D). Indeed, ~50% of the top 50 abundant transcripts associated with cluster 6 carry REPs (Table 1).

**TABLE 1:** Top 50 upregulated transcripts from cluster 6 (70 mM NaF versus control)

	Gene ID	Log_2_ fold change, (70 mM NaF vs control)	*P* value, (*t* test)	REP name	No. of REPs
1	*proP*	2.62	0.02	REP326	1
2	*ilvX*	2.44	0.03	REP285	1
3	*exbB*	2.41	0.06	REP222	5
4	*mgtA*	2.41	0.01	REP339	2
5	*otsB*	2.3	0		
6	*ilvC*	2.28	0.01		
7	*puuD*	2.21	0.01	REP112	2
8	*ycfJ*	2.19	0.02	REP103	1
9	*slyB*	2.18	0		
10	*osmC*	1.9	0.01	REP122	1
11	*exbD*	1.87	0.01	REP222	5
12	*sodA*	1.85	0		
13	*puuC*	1.84	0	REP112	2
14	*ldtE*	1.84	0		
15	*osmB*	1.82	0		
16	*puuR*	1.79	0.01	REP112	2
17	*yghA*	1.77	0.03	REP222	5
18	*ybjX*	1.75	0.02		
19	*mgtS*	1.72	0.01		
20	*puuA*	1.72	0.04	REP111	1
21	*otsA*	1.69	0.01		
22	*puuP*	1.64	0	REP111	1
23	*clpA*	1.62	0.07		
24	*rstA*	1.61	0.05		
25	*bdm*	1.61	0		
26	*ilvE*	1.58	0.02	REP285	1
27	*yrbL*	1.57	0.04		
28	*ytjA*	1.56	0		
29	*osmY*	1.55	0.03		
30	*aroF*	1.55	0.01	REP190	1
31	*tyrA*	1.46	0.02	REP190	1
32	*bax*	1.44	0.07	REP268	4
33	*modF*	1.44	0.18	REP71	1
34	*ilvM*	1.43	0.08	REP285	1
35	*fhuF*	1.42	0.05		
36	*ilvD*	1.4	0.07	REP285	1
37	*yhbO*	1.38	0.08		
38	*alaC*	1.38	0.07		
39	*phoQ*	1.37	0		
40	*betT*	1.37	0.04		
41	*amtB*	1.35	0.09	REP42	1
42	*dacC*	1.31	0.04	REP81	1
43	*glpD*	1.31	0.04	REP256	2
44	*ilvB*	1.28	0.52		
45	*pgpC*	1.26	0.05		
46	*betA*	1.26	0.03		
47	*cysQ*	1.24	0.08		
48	*ycgB*	1.21	0.13		
49	*proV*	1.21	0.04	REP193	2
50	*clsB*	1.18	0.03		

### (ii) Defining the major cellular processes and metabolic pathways affected by NaF treatment.

Our analysis of the gene expression clusters revealed principle categories of genes in E. coli that are up- or downregulated by NaF in a dose-dependent manner under anaerobic conditions (Fig. 3 and Table 2). We found that the upregulated genes are linked to controlling cell envelope stress (*pspABCDE* operon), osmotic stress adaptation (the *betIBA*, *proVWX*, and *otsBA* operons, as well as *proP*, *betT*, *treF*, *osmC*, *osmY*, *osmB*, and *ybaY* genes), signal transduction systems (the *phoPQ*, *rstAB*, and *yeaGH* operons), metabolism (the *ilvXGMEDA* and *ivbL-ilvBN* operons, as well as *ilvC*, *aroG*, and *aroF* genes), lipid biosynthesis (*ybhP*, *cslB*, *ybhN*, and *pgpC* genes), amine and polyamine degradation (the *puuDRCBE* operon), acquisition of iron (the *entCEBA*, *exbBD*, and *feoABC* operons, and the *yqjH* and *fhuF* genes), and iron homeostasis (*sufABCDSE* operon). In contrast, downregulated genes were involved in glycolysis (*gapA*, *yeaD*, *tpi*, and *fbp* genes), fatty/amino acids metabolism (the *argCBH*, *carAB*, and *rfbACX* operons, and the *accA*, *fabF*, *fabZ*, f*abB*, *argF*, *argA*, *argG*, *argI*, *metA*, *metB*, and *metK* genes), energy production (*cydAB* operon), cytochrome *c* biogenesis (the *yajC*, *yidC*, *yidD*, and *dsbA* genes), protein translocation (*tatABC* operon), translation (*rplK*, *rplM*, *rpsI*, *rpmB-rpmG*, *rpmH*, *rnpA*, *rpsU*, *rpsT rplS*, *rplT*, *rpsO*, *rpmE*, *rpmF*, *rpmA*, *rplU*, *rplY*, *prmB*, and *rimO* genes), translation regulation (*infC*, *infA*, *rsfS*, *rmlH*, *rrf*, *prfC*, *efp*, *yhbY*, and *arfA* genes), protein folding/processing (*grpE*, *groS*, *degP*, and *prc* genes), transport (the *cusCFBA*, *cusRS*, *rbsDACBKR*, *mglBAC*, *artPIQM*, *metNIQ*, *gatABCD*, and *manXYZ* operons and the *artJ* gene), and RNA metabolism (the *guaBA*, *purHD*, *hflD*, *purMN*, *rseABC*, *glyVXY* operons, as well as the *dcd*, *nrdA*, *dut*, *slmA*, *pyrC*, *adk*, *purB*, *putT*, *purl*, *purF*, *guaC*, *udk*, *rpoE*, *rpoZ*, *hns*, *dusB*, *fis*, *nusG*, *dksA*, *crp*, *lrp*, *pdhR*, *btsR*, *mraZ*, *ssrS*, *pcnB*, *rnc*, *era*, *rppH*, *yicC*, *ndk-rlmN*, *rsmA*, *rlmH*, *rlmI*, *rluC*, *rluE*, *rlhA*, *trmA*, *ppnP*, *cysS*, *asnS*, *glnS*, *proS*, *argS*, *tyrS*, *mnmA*, *mnmG*, and *rph* genes), including those coding for tRNA precursors (*valUXY-lysV*, *serV-argVYZQ*, *lysT-valT-lysW-valZ-lysY-lysQ*, *metT-LeuW-glnUW-metU-glnVX*, *thrU-tyrU-glyT-thrT*, *glyW-cysT-leuZ*, *aspT*, *serX*, *tyrTV*, *aspU*, *rrsA-ileT-alaT-rrlA-rrfA*, *rrsB-gltT-rrlB-rrfB*, *rrsC-gltU-rrlC-rrfC*, *rrsD-ileU-alaU-rrlD-rrfD-thrV-rrfF*, *rrsE-gltV-rrlE-rrfE*, *rrsG-gltW-rrlG-rrfG*, and *rrsH-ileV-alaV-rrlH-rrfH*).

**TABLE 2:** E. coli genes transiently upregulated (cluster 6) and downregulated (cluster 5) by NaF in a dose-dependent manner^*a*^

General categories	Operons	Functional subcategories	Transcriptional/translational regulators		Terminators
			Activator	Repressor	and REPs
Upegulated genes (Cluster 6)					
Cell envelope stress	*pspA* ***B*** *CD* ***E***	Inner membrane stress	RpoN		
Osmotic stress adaptation	*proP* (osmosensing transporter)	Uptake of zwitterionic osmolytes (l-proline:proton symport, glycine betaine:proton symport)	Fis, CRP, RpoS		REP326
	*betIBA, betT*	Biosynthesis of the osmoprotectant glycine betaine from choline	Cra, RpoS	BetI,	
				p-ArcA	
	*proV* ***WX***	Glycine betaine ABC transporter	IHF, RpoS	H-NS	REP193a-b
	*otsBA, treF*	Trehalose (osmoprotectant) metabolism	RpoS, ppGpp		
	*osmC*	Organic peroxidase activated by osmotic pressure	Na-NhaR	H-NS	REPt122
			pRcsB, ppGpp leu-Lrp		
	*osmY*	Periplasmic chaperone	ppGpp, RpoS	H-NS, CRP, Fis, IHF, FliZ, pArcA	
	*osmB*	Osmotic stress	RpoS, P-RcsB, Nac, ppGpp		
	(lipoprotein)				
	*ybaY*	Osmotic stress	FliA		
	(lipoprotein)				
Signal transduction systems	*phoPQ*	Phoqp Two-Component Signal Transduction System, magnesium-dependent	p-PhoP, RpoE	GcvB, MicA	
	*mgrB*	Phoq kinase inhibitor	PhoP		
	*rstAB*	Rstba Two-Component Signal Transduction System	PhoP		
	*yeaGH*	Putative yeagh Two-component	ppGpp, NtrC		REP134
		Signal transduction system			
Metabolism	*ilvX* ***G*** *MED* ***A***	Isoleucine	ppGpp	Lrp, GcvB	REP285
	*ivbL-ilvBN*	&	ppGpp, cAMP-CRP	GcvB	Rho-ind. terminator
	*ilvC*	Valine biosynthesis	IlvY		
	*ilvE* *alaC*	l-alanine biosynthesis II	ppGpp, InfA(B) SgrR	Lrp Nac	
	*aroG*, *aroF-tyrA*	3-dehydroquinate biosynthesis I	pCpxR	TyrR, Lrp	REP70
	*trpD* (anthranilate synthase subunit TrpD) *gltD* *ast****CAD****B****E***	l-glutamine degradation I / l-glutamate biosynthesis, l-tryptophan biosynthesis l-glutamine degradation I Ammonia assimilation cycle III l-glutamate biosynthesis I l-arginine degradation II (AST pathway)	SoxR TrpR, RydC Leu-Lrp, AdiY, RpoS, InfA, HdfR arg_ArgR p-NtrC, RpoS, RpoN	TyrR, Nac GcvB Fe^2+^-Fur, FNR, Nac, cAMP-CRP, Arg-ArgR Lrp, Rob	REPt190
	*talA-tktB*	Pentose phosphate pathway (nonoxidative branch)	pCreB, ppGpp, DksA		REP179a-c
	*maeA* *acnA*	Glyoxylate cycle, TCA cycle I (prokaryotic) mixed acid fermentation gluconeogenesis I Glyoxylate cycle / TCA cycle I	Cra, CRP, ppGpp, MarA, Rob, SoxS	FnrS pArc, Fnr	
	*ybdR*	Oxidoreductase		Nac	
	(putative alcohol dehydrogenase YbdR)				
Lipid biosynthesis	*pgpC*	Cardiolipin biosynthesis I	RpoH	NsrR	
	*ybhP-cslB-ybhN*	Cardiolipin biosynthesis I	ppGpp		
Amine and polyamine degradation	*puuDRCBE,*	Putrescine degradation II	RpoS	puuR, p-ArcA	REPv112a-b
				cAMP-CRP	
	*puuA*	Putrescine degradation II		puuR, p-ArcA, Fnr	
	*puuP*	Putrescine:H^+^ symporter puup	RpoN, RpoS	puuR, p-ArcA, Fnr	REPv111
Peptidoglycan maturation	*ldtE*	L, d-transpeptidase ldte	ppGpp		

	*dacC*		ppGpp	ArgR, pBolA	REPt81	
Acquisition of iron Metabolite damage control	*entCEBA*	Enterobactin biosynthesis	cAMP-CRP	Fe^2+^-Fur		
	*yqjH (nfeF)*	Adaptation to iron starvation		Fe^2+^-Fur, NfeR	5′ REP228a-b	
	*exbBD*	Transport of iron-siderophore complexes		Fe^2+^-Fur	REP222a-d	
					REPv222e	
	*feoABC*	Ferrous iron transport	Fnr, pOmpR	Fe^2+^-Fur, pArcA, NagC		
	*fhuF*	Ferric-siderophore reductase		Nac, Fe^2+^-Fur, OxyR		
	*efe* ***U*** *O* ***B*** *ybgl-pxpBC* ***A***	Ferrous iron transport system protein efeo 5-oxoprolinase		CpxR	REP95ab	
Iron homeostasis	*suf* ***A*** *B* ***C*** *D* ***SE***	Iron-sulfur cluster assembly	ppGpp, OxyR, IscR, OxyR, InfA	Fe^2+^-Fur,		
				NsrR		
Transcription factors	*mhpR*	Catabolism of aromatic compounds	cAMP-CRP			
	DNA-binding transcriptional activator MhpR					
DNA & protein repair	*yhbO*	Protein/nucleic acid deglycase 2	ppGpp	Rob		
Control of cell division and morphology	*fic* *drpB*	Cell filamentation induced by camp Cell division protein drpb	RpoS RpoE			
Miscellaneous	*modF*	Molybdenum uptake			REP71	
	*mgtA*	Transport of Mg^2+^	pPhoP		REP339a REPv339b	
	(Mg^2+^ importing P-type ATPase)					
	*mgtS*	Interacts with the Mg^2+^ transporter, mgta to promote intracellular Mg^2+^ accumulation Transport of ammonium	pPhoP, Nac GadX, pNtrC	Fnr, Fe^2+^-Fur	REPt42	
	(MgtS is a small, inner membrane protein) *amtB*					
	*ldcC*	Aminopropylcadaverine biosynthesis		GlaR		
	lysine decarboxylase 2	Cadaverine biosynthesis, l-lysine degradation I				
		
	*glpD*	Aerobic glycerol 3-phosphate dehydrogenase	cAMP-CRP	pArcA, GlpR, FnrS	REPv256a REP256b	
	*bdm*	Positive regulation of bacterial-type flagellum assembly	RcsB			
	(biofilm-dependent modulation protein)					
	*slyB* *clpA* *mlaF* ***EDCB***	Outer membrane lipoprotein slyb Clpa ATP-dependent protease specificity component and chaperone Intermembrane phospholipid transport system, ATP binding subunit mlaf	MarA	pPhoB	REP244	

	*sodA* (superoxide dismutase reduces iron toxicity)	Oxidative stress	SoxR, SoxS, cAMP-CRP, MarA, Rob	ArcA, Fe^2+^-Fur, Fur, InfAB FnrS, RyhB		
	*yghA*	NADP^+^-dependent aldehyde reductase	ppGpp	pBasR	REPt222a-e	

	*yrbL* *truB* *pepB* *bax* *yfdC* *yqjE*	Protein kinase-like domain-containing protein yrbl tRNA pseudouridine55 synthase Aminopeptidase B Putative glycoside hydrolase Bax Inner membrane protein yfdc Inner membrane protein yqje	PhoP, SoxS, SoxR Fis	Arg-ArgR cAMP-CRP GlaR	REP183b REP268a-d	
	*ytjA* *ybjX*	DUF1328 domain-containing protein ytja DUF535 domain-containing protein ybjx	YieP, FliA	Nac		
	*yldA*	Protein ylda				
	*ycfJ* *ycgB* *ymjE* *ybhG* *yobA-yebZ-yebY* *zntA* *fdoH*	PF05433 family protein ycfj PF04293 family protein ycgb Protein ymje Copd family protein Copper-sequestering Zn^2+^/Cd^2+^/Pb^2+^ exporting P-type ATPase Formate dehydrogenase O subunit β	Leu-Lrp ppGpp RpoS, RpoN ZntR	Nac, Lrp pArcA, Fnr, PuuR CecR sRNA FnrS sRNA SdhX	REPv111 REPv297b REPv297b	
Downregulated genes (Cluster 5)						
CENTRAL CARBON METABOLISM Glycolysis					
Gluconeogenesis I	*gapA-yeaD*	Glyceraldehyde-3-phosphate dehydrogenase gapa	cAMP-CRP, RpoE, RpoS RpoS	Cra	REPt133 REPt303	
Glycolysis II (from fructose 6-phosphate)	*ybhA*	Putative aldose 1-epimerase YeaD pyridoxal phosphate/fructose-1,6-bisphosphate phosphatase				
			
			
Glycolysis I (from glucose 6-phosphate) Pentose phosphate pathway (nonoxidative branch) I	*tpiA*	Triose-phosphate isomerase				
	*fbp*	Fructose-1,6-bisphosphatase 1				
	*tktA*	Transketolase 1				
						
TCA cycle	*sdhCD* ***AB***	Succinate dehydrogenase	Nac, cAMP-CRP, Fe^2+^-Fur	pArcA, pCpxR, Fnr, RyhB, Spf		
Glycerol degradation	*dhaKLM*		DhaR, RpoS		REPt107	
5-phosphoribosyl 1-pyrophosphate biosynthesis	*prs*	Ribose-phosphate diphosphokinas		PurR		
						
FATTY ACID METABOLISM						
Fatty acid biosynthesis initiation (type II)	*accA*	Acetyl-coa carboxyltransferase subunit α	FadR pCpxR, GadE, RpoE FadR			
	
	*fabF*					
	*fabZ*					
	*fabB*					
	*ispU*	Ditrans, polycis-undecaprenyl-diphosphate synthase ([2E,6E]-farnesyl-diphosphate specific)				
	*accA*	Acetyl-coa carboxyltransferase subunit α	FadR			
Lipoate biosynthesis and incorporation I	*yebD-lipB*	Lipoyl(octanoyl) transferase				
Phosphopantothenate biosynthesis I	*panB*	3-methyl-2-oxobutanoate hydroxymethyltransferase				
L-ornithine biosynthesis I	*argCBH*			argArgR argArgR argArgR, Lrp	REP207	
	*argF*					
	*argA*					
L-aspartate biosynthesis (+ 3 others)	*aspC*	Aspartate aminotransferase	Leu-Lrp	Fe^2+^-Fur, Fnr		
L-homoserine biosynthesis l-lysine biosynthesis I	*lysC*	Aspartate kinase III	ArgP			
	*dapB*	4-hydroxy-tetrahydrodipicolinate reductase	ArgP	Nac		
						
L-histidine biosynthesis	*hisG*	ATP phosphoribosyltransferase	ppGpp, DksA			
β-alanine biosynthesis III	*panD*	Aspartate 1-decarboxylase proenzyme		GcvB		
L-arginine biosynthesis I (via l-ornithine) Folate transformations III	*argG*		cAMP-CRP DksA, ppGpp pArcA, Fis, RutR	argArgR argArgR InfAB, PepA, RutR, argArgR SAM-MetJ		
	*argI*					
	
	*carAB* *metF*					
	
L-methionine biosynthesis I	*metA*		RpoE pPhoP	SAM-MetJ SAM-MetJ	REP306	
	*metB*					
S-adenosyl-l-methionine biosynthesis	*metK* *metJ*			SAM-MetJ Fe^2+^-Fur	REP215 REP305ab	
S-adenosyl-l-methionine salvage I						
dTDP-N-acetylthomosamine biosynthesis	*rfbACX*		GlaR	Nac		
dTDP-β-l-rhamnose biosynthesis	*rfbBD*					
CMP-3-deoxy-d-manno-octulosonate biosynthesis	*kdsA*	3-deoxy-d-manno-octulosonate 8-phosphate synthase	GlaR	Nac		
Acetate and ATP formation from acetyl-CoA I mixed acid fermentation ethanolamine utilization l-threonine degradation I	*ackA*	Acetate kinase	pArcA, pCreB, Fnr, Nac, RpoE	SdhX		
L-glutamine degradation I ammonia assimilation cycle III	*glsB*	Glutaminase 2				
						
Chorismate biosynthesis from 3-dehydroquinate	*aroK* ***B***	Shikimate kinase 1	ArgR			
						
Tetrahydromonapterin biosynthesis	*folX*	Dihydroneopterin triphosphate 2′-epimerase		FnrS		
Peptidoglycan maturation (meso-diaminopimelate containing)	*dacA*	D-alanyl-d-alanine carboxypeptidase daca				
	*rlpA*	Rare lipoprotein RlpA				
	*lptE*	Lipopolysaccharide assembly protein LptE				
	*murB*	UDP-*N*-acetylenolpyruvoylglucosamine reductase				
	*lpp*	Murein lipoprotein		pOmpR, MicL-S		
	*yafK*(*dpaA*)	Peptidoglycan meso-diaminopimelic acid protein amidase A	Nac			
	*mepS*	Peptidoglycan endopeptidase/peptidoglycan L, d-carboxypeptidase				
	*mipA*	MltA-interacting protein	pPhoB			
Peptidoglycan recycling I	*emtA*	Lytic murein transglycosylase E				
(Aminomethyl)phosphonate degradation	*ppa*	Inorganic pyrophosphatase				
						
ENERGY PRODUCTION						
						
	*cydA* ***B***	Cytochrome bd-I subunit 1	Nac, pArcA, Cra	H-NS, Fnr		
	*cydX*	Cytochrome bd-I accessory subunit CydX				
	*atpIB* ***EFHAGD***	ATP biosynthesis				
	*hybAO*	Hydrogenase 2 iron-sulfur protein		pNarL, pArcA		
	*ykgE*	Putative lactate utilization oxidoreductase YkgE	Leu-Lrp,	YieP, Nac		
	*adhE*	Fused acetaldehyde-coa dehydrogenase and iron-dependent alcohol dehydrogenase	Fis, Fnr, RpoS	Leu-Lpr, Cra, pNarL		
	*ldhA*	D-lactate dehydrogenase	RpoE, leu-Lrp			
	*aldA*	Aldehyde dehydrogenase A, NAD-linked	Mar, Rob, SoxS, cAMP-CRP	pArcA, DnaA, Fnr		
The SecYEG-SecDF-YajC-YidC holo-translocon (HTL) protein secretase/insertase						
cytochrome c biogenesis	*yajC*	Sec translocon accessory complex subunit YajC		ppGpp		
	*secG*	Sec translocon subunit SecG		Nac		
System I type	*yidC*	Membrane protein insertase YidC			REPv281	
	*yidD*	Membrane protein insertion efficiency factor				
	*dsbA*	Thiol:disulfide oxidoreductase DsbA	pCpxR	Nac		
Protein translocation	*tat* ***A*** *B* ***C***	Twin arginine protein translocation system-tatb protein				
Translation						
Ribosome proteins	*rplK*	50S ribosomal subunit protein L11		DksA, ppGpp, RplA		
	*rplM-rpsI*	50S ribosomal subunit protein L13, 30S ribosomal subunit protein S9	SrmB	cAMP-CRP, RplM, DksA, Fis, Fnr		
	*rpmB-rpmG*	50S ribosomal subunit protein L28, 50S ribosomal subunit protein L33		YfeC, DksA		
	*rpmH-rnpA*(*RNaseP*)	50S ribosomal subunit protein L34	Nac	YfeC, DksA ppGpp		
	*rpsU* *rpsT*	30S ribosomal subunit protein S21 30S ribosomal subunit protein S20		YfeC, DksA, LexA DksA		
	*rplS* *rplT*	50S ribosomal subunit protein L19 50S ribosomal subunit protein L20		DksA, Fnr RplT, DksA, Fnr, NsrR		
	*rpsO*	30S ribosomal subunit protein S15	Fis, RpsA	argArgR, ppGpp, cAMP-CRP, RpsO		
	*rpmE rpmF*	50S ribosomal subunit protein L31 50S ribosomal subunit protein L32	RpoH	ppGpp, RpmE		
	*rpmA, rplU*	50S ribosomal subunit protein L27	MlrI	DksA, ppGpp	REP243	
	*rplY*	50S ribosomal subunit protein L25		RplY, DksA, ppGpp	REP159	
	*prmB*	Ribosomal protein L3 N5-glutamine methyltransferase		Nac		
	*rimO*	Ribosomal protein S12 methylthiotransferase rimo				
Translation factors	*infC*	Translation initiation factor IF-3		DksA, NsrR		
	*infA*	Translation initiation factor IF-1		ppGpp, YeiE		
	*rsfS-rmlH*					
	*rrf* *frr*	Ribosome recycling factor Ribosome-recycling factor		ppGpp ppGpp		
	*prfC*	Peptide chain release factor RF3		ppGpp, Nac		
	*efp*	Protein chain elongation factor EF-P		ppGpp		
	*yhbY*	Ribosome assembly factor yhby		Leu-Lrp		
	*arfA*	Alternative ribosome-rescue factor A				
Regulation of translation	*csrB*	Small regulatory RNA csrb	DksA, ppGpp, pUvrY, InfAB			
						
Protein folding	*grpE*	Nucleotide exchange factor grpe	cAMP-CRP, RpoE			
	*groS*	Cochaperonin groes	Nac, RpoE,	Leu-Lrp		
	*degP* *hdeAB-yhiD*	Periplasmic serine endoprotease degp Energy-independent periplasmic chaperone	pCpxR GadY, pRcsB, pPhoB, pTor, ppGpp, RpoS	H-NS GadW, GadX, pLrp, FliZ, H-NS, MarA		
	*fkbA*	Peptidyl-prolyl cis-trans isomerase fkpa	ppGpp, RpoE			
	*ppiA*	Peptidyl-prolyl cis-trans isomerase A	pCpxR	CytR, cAMP-CRP		
	*narW*	Narw, putative private chaperone for narz nitrate reductase subunit	pOmpR			
Protein processing	*prc*	Tail-specific protease				
	*bepA*	Β-barrel assembly enhancing protease	RpoE			
TRANSPORT						
	*ompF*	Outer membrane porin F	cAMP-CRP, EnvY, Fe^2+^-Fur, pPhoB	InfBA, pOmpR, Nac, pCpxR, pRstA, MicF, RybB		
	*bamD*	Outer membrane protein assembly factor bamd	RpoE			
Cu^+^/Ag^+^ export	*cusCFBA*		pPhoB. pHprR, pCusR			
	*cusRS*	DNA-binding transcriptional activator cusr	pPhoB. pHprR, pCusR		REP261	
	*pitA*	Metal phosphate:H+ symporter pita	Fnr	MgrR		
	*rcnB*	Periplasmic protein involved in nickel/cobalt export	RpoE	RcnR, Fe^2+^-Fur		
Transport of K+	*kdp* ***F*** *A* ***BC***	Potassium ion importing Kdp atpase	pKdpE	pArcA		
						
	*dctA*	C4 dicarboxylate/orotate:H+ symporter	cAMP-CRP, DcuR	pArcA		
	*dauA*	Erobic C4-dicarboxylate transporter daua		YieP		
	*dppA*	Dipeptide ABC transporter periplasmic binding protein	pArcA, InfAB	Leu-Lrp, Nac, Fnr	REPv266b	
Transport of thiamine	*thiB* ***PQ***	Thiamine ABC transporter periplasmic binding protein	DksA, ppGpp	SgrR	REP7ab	
Transport of l-aspartate Transport of l-glutamate	*gltI*	Glutamate/aspartate ABC transporter periplasmic binding protein	FhlC-FhlD			
Transport of spermidine	*potD*	Spermidine preferential ABC transporter periplasmic binding protein				
Transport of d-ribopyranose	*rbsDACBK* ***R***		cAMP-CRP	YidZ, RbsR, DsrA		
Transport of d-galactopyranose	*mglBAC*		cAMP-CRP, RpoS	Nac, FlhCD, GalR, GalS	REP156abc	
L-arginine ABC transporter	*artJ*	Periplasmic binding protein	RpoS	argArgR leu-Lrp, ArgR	REP83a-b	
	*artPI* ***QM***	ATP binding subunit				
	*plaP*	Putrescine:H+ symporter plap				
	*lysP*	Lysine:H+ symporter	ArgP	Leu-Lrp, Nac		
	*dtpA*	Dipeptide/tripeptide:H+ symporter dtpa	pOmpR	Leu-Lrp, GadX		
L-methionine/d-methionine ABC transporter Exporters	*metN* ***I*** *Q* *alaE*	l-alanine exporter	GlaR, Leu-Lrp,	SAM-MetJ		



	*yohJ*	Putative 3-hydroxypropanoate export protein yohj		YieP		
PTS systems						
	*ptsHI-crr*	Phosphocarrier protein hpr		Cra, cAMP-CRP, Mic, NagC		
	*yeeX(tmaR)*	PTS system regulator tmar				
Galactitol-specific PTS enzyme	*gatABCD*	Galactitol-specific PTS enzyme	cAMP-CRP	Nac, pArcA, GatR		
Mannose-specific PTS enzyme	*manXY* ***Z***	Mannose-specific PTS enzyme	cAMP-CRP	Cra, Mlc, NagC, DicF, SgrS		
	*ptsG*	Glucose-specific PTS enzyme IIBC component	cAMP-CRP, SoxS	Fis, pArcA, Mlc		
Xanthine:proton symport	*xanP*	Xanthine:H+ symporter xanp				
						
Redox proteins / systems	*trxB*	Thioredoxin reductase (NADPH)		Nac, Leu-Lrp		
	*grxD*	Glutaredoxin 4		NsrR, RyhB	REPt127	
	*bcp*	Thiol peroxidase				
	*tpx*	Lipid hydroperoxide peroxidase		pArcA, Fnr		
STRESS RESISTANCE	*hdeD*	Acid resistance membrane protein	GadE, GadX, pRcsB, pPhoP, ppGpp, RpoS	H-NS		
	*yqgB*	Acid stress response protein yqgb	Nac			
	*yfgG*	Nickel/cobalt stress response protein yfgg				
	*ychF*	Redox-responsive atpase ychf		OxyR, ppGpp		
	*sodB*	Superoxide dismutase (Fe)		cAMP-CRP, InfAB, NsrR, FnrS, RyhB	REPv128ab	
	*opgG*	Osmoregulated periplasmic glucans biosynthesis protein G	RpoE			
	*uspF*	Universal stress protein F	YeiE			
	*ydiY*	Acid-inducible putative outer membrane protein ydiy		Nac		
						
						
SIGNAL TRANSDUCTION SYSTEMS	*rcsF*	Sensor lipoprotein rcsf				
	*dgcP*	Diguanylate cyclase dgcp				
						
CELL DIVISION/RECOMBINATION/DNA REPLICATION						
	*cpoB*	Cell division coordinator cpob		Leu-Lrp, MicA		
	*ftsB*	Cell division protein ftsb				
	*mreB*	Dynamic cytoskeletal protein mreb		pBolA		
	*mioC* *rdgC*	Flavoprotein mioc Nucleoid-associated protein rdgc		AsnC, MraZ, Nac, ppGpp		
	*holE*	DNA polymerase III subunit θ		Nac		
						
						
RNA AND DNA METABOLISM						
Pyrimidine deoxyribonucleotides *de novo* biosynthesis II	*dcd*	Dctp deaminase	RbsR			
	*nrdA*	Ribonucleoside-diphosphate reductase 1, α subunit dimer	Fis, ArgP, cAMP-CRP	NrdR, H-NS, DnaA	REP164a-e	
	*dut-slmA*	Dutp diphosphatas			REPt275	
UMP	*pyrC*	Dihydroorotase		PurR, Fe^2+^-Fur		
biosynthesis I						
Guanosine ribonucleotides *de novo* biosynthesis	*guaB* ***A***	Inosine 5′-monophosphate dehydrogenase	cAMP-CRP	PurR, Fis, DnaA		
Guanine and guanosine salvage III Adenine and adenosine salvage V	*gsk*	Inosine/guanosine kinase			REP46ab	
	*gpt*	Xanthine-guanine phosphoribosyltransferase		Leu-Lrp		
UTP and CTP *de novo* biosynthesis	*adk*	Adenylate kinase			REPt45	
Inosine-5′-phosphate biosynthesis I	*purHD*		pPhoP	PurR, RbsR		
	*hflD-purB*	Lysogenization regulator, adenylosuccinate lyase		PurR	REPt106	
5-aminoimidazole ribonucleotide biosynthesis II	*purT*	Phosphoribosylglycinamide formyltransferase 2	Lrp	PurR	REPt136	
	*purL*	Phosphoribosylformylglycinamide synthetase		PurR		
	*purF*	Amidophosphoribosyltransferase		PurR		
	*pur* ***M*** *N*	Phosphoribosylglycinamide formyltransferase 1		PurR, Fnr		
Interconversion of nucleotides	*guaC*	GMP reductase				
	*udk*	Uridine/cytidine kinase	RbsR			
TRANSCRIPTION	*rpoE*	RNA polymerase sigma factor rpoe (sigma 24 factor)	cAMP-CRP, DksA, GlrR, NtrC, RcsB, ppGpp, RpoN, RpoE, RpoS	pCpxR, InfAB		
	*rpoZ*	RNA polymerase subunit ω		Nac, DksA		
					
	*hns*	Transcription factor	CspA, Fis, GadX	H-NS, ppGpp, DsrA		
	*dusB-fis*	Transcription factor Fis	InfAB, cAMP-CRP	DksA, ppGpp, Fis		
	*nusG*	Transcription termination / antitermination factor nusg		OxyS		
	*cspE*	Transcription antiterminator and regulator of RNA stability cspe	cAMP-CRP	DinJ-YafQ		
	*yeiP*	Elongation factor P-like protein yeip	cAMP-CRP			
	*dksA*	RNA polymerase-binding transcription factor dksa	cAMP-CRP	DksA, ppGpp		

	*crp*	DNA-binding transcriptional dual regulator CRP	Cra, cAMP-CRP	ppGpp, Fis		
	*rseAB* ***C***	Anti-sigma-E factor rsea	cAMP-CRP, DksA, GlrR, NtrC, RcsB, ppGpp, RpoN, RpoE, RpoS	pCpxR, InfAB		
	*idlP*	*Irad leader peptide*	ppGpp, Fnr	DksA, DnaA		
	*lrp*	DNA-binding transcriptional dual regulator Lrp	ppGpp, GadE	Lrp, H-NS, Nac, NsrR, ArgR, GcvB, MicF, DsrA		
	*pdhR*	DNA-binding transcriptional dual regulator pdhr	pOmpR, GlaR, cAMP-CRP, RpoS	Fnr, Cra, PdhR, pBtsR		
	*btsR*	DNA-binding transcriptional dual regulator btsr				
	*mraZ*	DNA-binding transcriptional repressor mraz		MraZ, PdhR		
	*marA*	DNA-binding transcriptional dual regulator mara	cAMP-CRP, pCpxR, Fis, MarA, Rob, SoxS	AcrR, Cra		
	*dicA*	DNA-binding transcriptional dual regulator dica	DicA			
	*narP*	DNA-binding transcriptional dual regulator narp	RpoE	RprA, SdsN		
	*hupA*	Transcriptional dual regulator HU-α (HU-2)	Nac, cAMP-CRP, Fis			
	*hupB*	DNA-binding protein HU-β	cAMP-CRP	Fis		
	*adiY*	DNA-binding transcriptional activator adiy	H-NS	SgrS		
	*uvrY*	DNA-binding transcriptional activator uvry	DeaD	Nac, LexA		
	*ssrS*	6s rna	Fis, RpoS	H-NS, Lrp, StpA, 6S RNA		
RNA turnover	*pcnB*	Poly(A) polymerase I		DksA, ppGpp		
	*rnc-era*	Ribonuclease III				
	*rppH*	RNA pyrophosphohydrolase				
	*yicC*	Putative RNase adaptor protein yicc				
tRNA and rRNAs	*valUXY-lysV*		Fis	B2401		
	*serV-argVYZQ*			B2695		
	*lysT-valT-lysW-valZ-lysY-lysQ*		Fis	B0743		
	*metT-LeuW-glnUW-metU-glnVX*		Fis	Lrp, ppGpp, b0673 ppGpp	REP60ab	
	*thrU-tyrU-glyT-thrT*		Fis Fis	ppGpp ppGpp		
	*glyVXY*					
	*glyW-cysT-leuZ*					
	*aspT, serX*					
	*tyrTV*		Fis	ppGpp		
	*aspU* *lysZ*	Trna-Lys(UUU)				
	*hisR*	Trna-His(GUG)	DksA, Fis	DksA-ppGpp		
	*aspU*	Trna-Asp(GUC)				
	*rrs****A***-*ileT-ala****T***-*rrl****A***-*rrf****A***	Ribosomal RNA operon A		DksA, ppGpp	REP292	
	*rrs****B***-*gltT-rrl****B***-*rrf****B***	Ribosomal RNA operon B	Fis, RpoE	DksA, H-NS		
	*rrs****C***-*gltU-rrl****C***-*rrf****C***	Ribosomal RNA operon C	Fis, RpoE	DksA, H-NS		
	*rrs****D***-*ileU-alaU-rrl****D***-*rrf****D****-thr****V***-*rrf****F***	Ribosomal RNA operon D	Fis, RpoE	DksA, H-NS		
	*rrs****E***-*gltV-rrl****E***-*rrf****E***	Ribosomal RNA operon E	Fis, RpoE	DksA, H-NS		
	*rrs****G***-*gltW-rrl****G***-*rrf****G***	Ribosomal RNA operon G	Fis, RpoE	DksA, H-NS		
	*rrs****H***-*ileV-alaV-rrl****H***-*rrf****H***	Ribosomal RNA operon H	Fis, RpoE	DksA, H-NS		
RNA modifying enzymes	*ndk-rlmN*			pArcA		
	*rsmA*	16S rrna m^6^_2_a1518, m^6^_2_a1519 dimethyltransferase	Fis, RpoE		REP5	
	*rlmH*	23S rrna m^3^ψ1915 methyltransferase				
	*rlmI*	23S rrna m^5^c1962 methyltransferase				
	*rluC*	23S rrna pseudouridine^955/2504/2580^ synthase		ppGpp		
	*rluE*	23S rrna pseudouridine^2457^ synthase	Nac			
	*rlhA*	23S rrna 5-hydroxycytidine C2501 synthase				
	*trmA*	Trna m5u54 methyltransferase	Fis, RpoH			
	*ppnP*	Nucleoside phosphorylase ppnp				
	*proQ*	RNA chaperone proq				
tRNA Ligases	*cysS*	Cysteine—trna ligase		GlaR, Nac	REPt53	
	*asnS*	Asparagine—trna ligase				
	*glnS*	Glutamine—trna ligase	RpoH			
	*proS*	Proline—trna ligase				
	*argS*	Arginine—trna ligase		Leu-Lrp, ppGpp		
	*tyrS*	Tyrosine—trna ligase				
	*mnmA*	Trna-specific 2-thiouridylase		leu-Lrp		
	*mnmG* *metG*	5-carboxymethyl-aminomethyluridine-trna synthase subunit mnmg Methionine—trna ligase	RpoS	AsnC, Nac, ppGpp		
tRNA maturation	*rph*	Truncated RNase PH				
	*suhB*	Nus factor suhb				
						
Miscellaneous	*ybgE* *ybgC-tolQ-TolR* *ycaC*	PF09600 family protein ybge Tol-Pal system Putative hydrolase ycac	Nac pBaeR	Nac Fnr, Nac		
	*ycaD*	Putative transporter ycad		Nac		
	*ybhC*	Outer membrane lipoprotein ybhc			REP72abcd	
	*yajQ*	Nucleotide binding protein yajq			REP40a	
	*tamAB-ytfP*					
	*ytfP*	Γ-glutamylamine cyclotransferase family protein ytfp		Nac		
	*yhdV*	Lipoprotein yhdv	Leu-Lrp	Nac		
	*yajG*	Putative lipoprotein yajg				
	*ytiD*	Protein ytid	ppGpp	DksA, DnaA		
	*yidB*	DUF937 domain-containing protein yidb	GlaR			
	*coaA*	Pantothenate kinase / pantetheine kinase				
	*rclB*	DUF1471 domain-containing protein rclb	RclR, RpoE	CsrA		
	*yqhA*	UPF0114 family protein yqha				
	*yagN*	CP4-6 prophage; protein yagn				
	*yqgC*	Protein yqgc				
	*glgS*	Surface composition regulator	Rob, ppGpp, cAMP-CRP			
	*yfcD*	Putative Nudix hydrolase yfcd		ArgR, PurR		
	*cvpA*	Colicin V production protein				
	*sixA*	Phosphohistidine phosphatase sixa				
	*yfdH*	CPS-53 (kple1) prophage; bactoprenol glucosyl transferase				
	*yfhL*	Putative 4Fe-4S cluster-containing protein yfhl		Nac		
	*ratA*	Ribosome association toxin rata				
	*rnlA*	CP4-57 prophage; RNase LS, toxin of the rnlab toxin-antitoxin system		IscR		
	*yecN*	MAPEG family inner membrane protein yecn				
	*ftnA*	Ferritin iron-storage complex	Fe^2+^-Fur	H-NS		
	*ftnB*	Putative ferritin-like protein	pCpxR, Nac			
						
	*yecJ*	DUF2766 domain-containing protein yecj				
	*yedL*	Putative acetyltransferase yedl		Nac		
	*mtfA*	Mlc titration factor		Nac		
	*ymiA*	Uncharacterized protein ymia		YjjQ		
	*yciX*	Uncharacterized protein ycix				
	*ldrB*	Small toxic polypeptide ldrb				
	*ldrC*	Small toxic polypeptide ldrc				
	*ymjA*	DUF2543 domain-containing protein ymja		Nac		
	*yhaM*	Rac prophage; protein ynam				
	*ynfS*	Qin prophage; protein ynfs				
	*ydiH*	Uncharacterized protein ydih				
	*yoaJ* *yobB*	Uncharacterized protein yoaj Putative carbon-nitrogen hydrolase family protein yobb		Nac, pOmpR		

a The genes in cluster 5 and 6 that were insignificantly affected by NaF are in bold. Transcriptional/translational regulation and presence of REP-elements are indicated according to the EcoCyc database (https://ecocyc.org/). The global regulators Fis, Crp, Lrp, H-NS, and RpoE are indicated by gray shading.

### Northern blot validation of altered transcript expression levels in response to NaF treatments.

Our tiling array data revealed that the abundance of numerous RNAs increased in a dose-dependent manner in response to NaF treatment (0 → 20 → 70 mM NaF), and subsequently decreased upon dilution of the NaF-treated cultures. To validate this expression pattern, we assessed the levels of several transcripts—namely, *osmC*, *proP*, *efeO*, and *yghA*—by means of Northern blot analysis. Consistently, we found that levels of all four transcripts increased upon NaF treatment (Fig. 3Ca–d; compare lines 1, 2, and 3), and their abundance subsequently decreased following dilution of the NaF-treated cultures (Fig. 3Ca–d; compare lines 4, 5, and 6). We selected *osmC* and *yghA* as representatives for further analysis as model mRNA species, because of following features: (i) both transcripts are in the top list of upregulated transcripts upon NaF treatment (Table 1); (ii) these transcripts carry different numbers of REPs (*osmC* and *yghA* carry 1 and 5 REPs, respectively), thus allowing us to investigate if less or more REPs can alter RNA decay; and (iii) their abundance was sufficiently high for REP detection by Northern blotting using specific probes.

As the impacts of increased salinity (NaCl) on gene expression have been studied previously (23, 24), we have referred to these studies to address whether NaCl (70 mM) has impact on gene expression of *osmC*, *proP*, *efeO*, and *yghA*. Our experimental data (Fig. S1; compare lines 1 and 2) support the conclusion that the increased levels of the above transcripts are mainly due to exposure to fluoride rather than due to a general increase in osmolarity.

### Degradation of REP-containing *osmC* and *yghA* mRNAs depends on RNase E and is impaired by NaF treatment.

**FIG 4:**
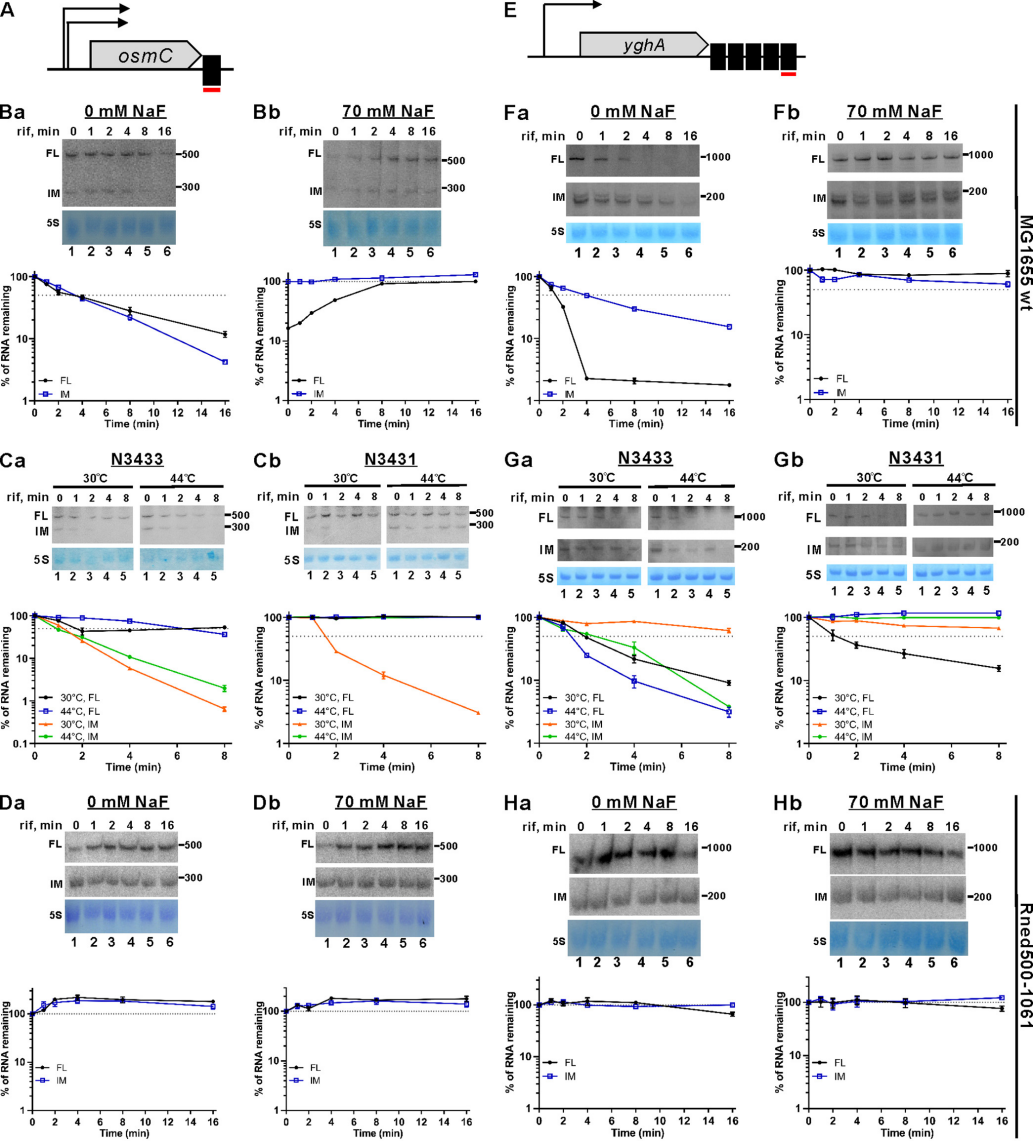
Stabilization of REP-containing full-length *osmC* and *yghA* mRNAs and their decay intermediates upon treating E. coli with NaF, inactivating RNase E, or deletion of the Rne C-terminal region. (A and E) Schematic representations of the *osmC* and *yghA* operons, respectively. REPs are depicted as black rectangles. The region complementary to the specific probe is indicated by a thin red line under the REPs. (B) Inhibition of ATP-dependent *osmC* RNA decay upon NaF treatment in MG1655 wt strain. Northern blot analysis was used to determine the half-lives of full-length *osmC* (FL) and its intermediate degradation product (IM) in the absence (0 mM) or presence (70 mM) of NaF under anaerobic conditions. The calculated half-lives were 5.4 ± 0.04 (FL) and 3.5 ± 0.05 (IM) min for control samples (0 mM NaF), and >16 min for both FL and IM RNAs in cells treated with 70 mM NaF. (C) Effect of RNase E inactivation on E. coli *osmC* stability. Experiments were conducted on temperature-sensitive (N3431) and isogenic wild-type (N3433) E. coli strains at permissive (30°C) and nonpermissive (44°C) temperatures under anaerobic conditions. Equal amounts of total RNA extracted from the cells before (control) and after rifampicin treatment at the times indicated at the top of each lane were analyzed by Northern blotting. The RNA half-lives determined for N3433 were >8 min (FL) and 1.1 ± 0.02 min (IM) at 30°C or 5.5 ± 0.01 min (FL) and 1.4 ± 0.03 min (IM) at 44°C, respectively. Northern blots on RNA extracted from N3431 revealed RNA half-lives of >8 min (FL) and 1.5 ± 0.02 min (IM) at 30°C or >8 min for both FL and IM RNAs at 44°C, respectively. RiboRuler RNA Ladders (Thermo Scientific) were used as size markers. The graphs below each blot show the relative abundance (mean value) of each RNA, whereas the vertical bar corresponds to the standard deviation. The dotted gray line corresponds to 50% (Ba, Ca, Cb) or 100% (Bb) of total RNA remaining. (F) Inhibition of ATP-dependent *yghA* RNA decay upon NaF treatment. Northern blot analysis was used to determine the half-lives of full-length *yghA* (FL) and its intermediate degradation product (IM) in the absence (0 mM) or presence (70 mM) of NaF under anaerobic conditions. The calculated half-lives were 2.8 ± 0.03 (FL) and 6.2 ± 0.12 (IM) min for control samples (0 mM NaF) or >16 min for both FL and IM RNAs in cells treated with 70 mM, respectively. (G) Effect of RNase E inactivation on E. coli *yghA* RNA stability. Experiments were conducted on temperature-sensitive (N3431) and isogenic wild-type (N3433) E. coli strains at permissive (30°C) and nonpermissive (44°C) temperatures under anaerobic conditions. Equal amounts of total RNA extracted from the cells before (control) and after rifampicin treatment at the times indicated at the top of each lane were analyzed by Northern blotting. The RNA half-lives determined for N3433 were 2.2 ± 0.12 min (FL) and >8 min (IM) at 30°C or 1.6 ± 0.12 min (FL) and 1.7 ± 0.06 min (IM) at 44°C, respectively. Northern blots of RNA extracted from N3431 revealed RNA half-lives of 3.3 ± 0.08 min (FL) and >8 min (IM) at 30°C or >8 min for both FL and IM RNAs at 44°C, respectively. RiboRuler RNA ladders (Thermo Scientific) were used as a size marker. The graphs beneath each blot show the relative abundance (mean values) of each RNA, whereas vertical bars correspond to standard deviations. The dotted gray line corresponds to 50% of total RNA remaining. (D and H) Degradosome-dependent *osmC* and *yghA* RNA decay in the Rned500-1061 strain. Northern blot analysis was used to determine the half-lives of full-length (FL) and its intermediate degradation product (IM) in the absence (0 mM) or presence (70 mM) of NaF under anaerobic conditions. The calculated half-lives were >16 min for both FL and IM RNAs in cells nontreated or treated with 70 mM NaF. RiboRuler RNA ladders (Thermo Scientific) were used as size markers. The graphs below each blot show the relative abundance (mean value) of each RNA, whereas the vertical bar corresponds to the standard deviation. The dotted gray line corresponds to 100% of total RNA remaining.

To determine if *osmC* and *yghA* transcript accumulation is posttranscriptionally regulated, we employed Northern blot analyses to measure the stability of *osmC* and *yghA* mRNAs in the presence or absence of NaF. As shown in Fig. 4Ba and Bb and 4Fa and Fb, exposing E. coli to 70 mM NaF inhibited RNA decay of the full-length (FL) *osmC* (~500 nucleotides, nt) and *yghA* (~1,000 nt) transcripts, as well as their decay intermediates (IM; fragments of ~200 nt and ~300 nt, respectively). More specifically, the experimentally determined half-lives of *osmC* mRNA and its decay intermediate in untreated (0 mM NaF) E. coli (i.e., 5.4 ± 0.04 [FL] and 3.5 ± 0.05 [IM], respectively) increased upon exposure to 70 mM NaF, reaching >16 min for both FL and IM RNAs. Similarly, the half-lives of *yghA* RNA transcripts (i.e., 2.8 ± 0.03 min [FL], 6.2 ± 0.12 min [IM]) in untreated (0 mM NaF) E. coli were considerably shorter than those following exposure to 70 mM NaF (i.e., >16 min). These results demonstrate that upregulation of *osmC* and *yghA* upon NaF treatment is exerted at the posttranscriptional level. To investigate the mechanisms potentially involved in degrading these RNAs, we explored if RNase E is required for *osmC* and *yghA* RNA degradation. To do so, we compared their transcript stability in wild-type E. coli N3433 and in RNase E temperature-sensitive strain N3431 (25), in which RNase E activity is inhibited under nonpermissive temperatures, thereby leading to RNase E-dependent transcript accumulation at nonpermissive temperatures solely in N3431. Thus, RNase E-dependent transcripts could be identified by comparing the corresponding Northern blot signals obtained at a permissive (30°C) versus nonpermissive (44°C) temperature. We observed that *osmC* transcripts accumulated in the N3431 strain (Fig. 4Cb; compare left and right), and their half-lives increased at 44°C (i.e., >8 min for both FL and IM RNAs) relative to at 30°C (>8 min [FL], 1.5 ± 0.02 min [IM]), indicative of RNA stabilization. In contrast, such stabilization was not observed for the wild-type strain (isogenic to N3431) (Fig. 4Ca; compare left and right) at 30°C (>8 min [FL], 1.1 ± 0.02 min [IM]) versus 44°C (5.5 ± 0.01 min [FL], 1.4 ± 0.03 min [IM]). Likewise, we detected no increase in stability of *yghA* transcripts in N3433 (Fig. 4Ga), in which RNA half-lives were 2.2 ± 0.12 min (FL) or >8 min (IM) at 30°C and 1.6 ± 0.12 min (FL) or 1.7 ± 0.06 min (IM) at 44°C, respectively. In contrast, stabilization of full-length and intermediate *yghA* mRNAs was observed in the temperature-sensitive N3431 strain (Fig. 4Gb), in which half-lives were 3.3 ± 0.08 min (FL) or >8 min (IM) at 30°C and >8 min for both FL and IM RNAs at 44°C. Thus, the higher stabilities of the *osmC* and *yghA* transcripts upon inactivation of RNase E indicate that their degradation is RNase E-dependent.

In addition, experiments were conducted on Rned500-1061 mutant (19) coding for C-terminal-truncated RNase E (1 to 499 aa; see Fig. 4D and H). This polypeptide contains only the catalytic domain and lacks the scaffolding region involved in interacting with major degradosome components, including PNPase, RhlB helicase and enolase (26, 27).

Our data show that *osmC* and *yghA* mRNAs are stabilized (half-lives >16 min; Fig. 4Da and 4Ha) even in the absence of NaF, supporting that degradation of the FL and IM mRNAs is degradosome-dependent. We also demonstrate that maximal stabilization of these transcripts is achieved in the absence of fluoride ions, so addition of NaF does not elicit further increases in transcript levels (Fig. 4; compare 4Da and Db, and 4Ha and Hb).

Collectively, our data indicate that the levels of the *osmC* and *yghA* transcripts are greatly impacted by RNase E/degradosome-dependent degradation (Fig. 4C and G). Namely, FL and IM transcripts of *osmC* and *yghA* mRNAs are stabilized in an RNase E-temperature sensitive strain and RNase E mutant coding for C-terminal-truncated (500 to 1,061 aa deleted) enzyme (half-lives >8 to 16 min; Fig. 4C and G). In other words, in RNase E thermosensitive and mutant strains, we observe accumulation/stabilization of these transcripts.

## DISCUSSION

Fluorine is ubiquitous on Earth. Its ion (fluoride) plays a vital role in many organisms, including humans (4). Clinical studies have revealed the physiological range of fluoride concentrations and demonstrated that insufficient or excessive fluoride intake causes various diseases (28). At high concentrations, fluoride acts as an antimicrobial agent that can inhibit bacterial growth and metabolic activities (20). Therefore, using fluoride-containing toothpaste or other anticavity drugs should impact the composition and function of the oral and gut microbiomes. Recent studies have shown that fluoride exerts its inhibitory effects on bacterial metabolism in many ways (4, 29). For instance, it can act as an enzyme inhibitor by interacting with the various metal ions present in many phosphatases, kinases, hydrolases, and other metalloproteins, or as an enhancer of membrane permeability by increasing proton uptake, thereby inducing cytoplasmic acidification. Most studies on fluoride-mediated enzymatic inhibition have focused on eukaryotic enzymes, especially those of yeast. Very similar inhibitory effects have since been observed for Streptococcus sp. (30) and other bacteria. However, the antibacterial actions of fluoride appear to be complex and remain incompletely understood.

One of the best-known protein targets of fluoride is enolase (6, 31). This enzyme catalyzes the penultimate step of glycolysis by converting 3-phosphoglycerate to phosphoenolpyruvate (5). Fluoride-mediated inhibition of enolase and phosphoenolpyruvate synthetase blocks glycolysis, thereby potentially depleting ATP. Indeed, NaF treatment of eukaryotic cells has been found to reduce ATP levels (8). Although diminished ATP levels can have profound effects on gene expression at the transcriptional and posttranscriptional levels, especially in animal microbiota under microaerobic conditions, the actual consequences of fluoride on gene expression in bacteria had been unclear.

Here, we used E. coli MG1655 grown under anaerobic conditions to mimic the environment of the dental plaque gastrointestinal tract, allowing us to study the impact of NaF-mediated ATP depletion on gene expression at the whole-transcriptome level as well as RNA decay. We found that ATP levels gradually declined in response to E. coli being exposed to elevated concentrations of NaF under anaerobic conditions (Fig. 1A). Despite inhibited growth, the E. coli cells retained their filamentous morphology (i.e., the typical appearance of anaerobically grown E. coli cells [19]) upon treatment with moderate (20 mM) or high (70 mM) NaF concentrations, and the integrity of control (untreated) and treated cells was almost indistinguishable (Fig. 1B).

As E. coli adapts to altered oxygen availability and/or upon exposure to molecules/chemicals such as NaF, it typically adjusts its metabolism by reprogramming gene expression (32, 33), resulting in changes in steady-state levels of mRNAs that are determined by rates of transcription and decay. Tiling microarrays (34) represent a valuable tool for establishing the whole-genome transcriptomes of microbial organisms exposed to various stress conditions. Here, we used such an array built at 25 nt resolution to observe changes in E. coli MG1655 gene expression upon NaF treatment. We anticipated that NaF-mediated ATP depletion would result in downregulated expression of genes involved in ATP-dependent processes. Nevertheless, we also expected that a subset of genes whose transcripts are stabilized upon NaF-associated inhibition of their ATP-dependent RNA turnover would be upregulated. NaF-dependent cell growth inhibition has previously been reported as reversible, both *in vitro* and *in vivo* (9, 10), following dilution of the NaF-treated cultures with fresh media lacking this reagent. To explore that scenario further, first we inhibited enolase activity by means of dose-dependent NaF treatments (i.e., at 0, 20 and 70 mM NaF; Fig. 1C). Then, we diluted the NaF-treated cultures (Fig. 1C) with the intention to reverse the changes in gene expression elicited by NaF. Next, we isolated RNA from each of the cultures and hybridized it to tiling array. Using a quantile-based K-means clustering approach on the tiling array data set, we identified two clusters (5 and 6) comprising genes whose expression changed in a dose-dependent manner upon NaF treatment (Fig. 2). Our data revealed that the 341 genes in cluster 5 were first downregulated and then upregulated following NaF treatment and subsequent dilution of the NaF-treated cultures, respectively. In contrast, the 100 genes in cluster 6 were initially upregulated and then downregulated upon NaF treatment and subsequent dilution.

An increase in expression of the *talA-tktB* operon controlling the pentose phosphate pathway indicates that the fluoride-dependent inhibition of glycolysis due to the negative effect of fluoride on glycolytic enzymes induces E. coli to increase glucose catabolism via alternative routes such as the pentose phosphate pathway.

Moreover, another group of upregulated genes involves those controlling metal ion transport. As fluoride can associate and form stable complexes/precipitates with a number of metals (e.g., calcium, magnesium, and iron) in bacteria and other organisms (4), exposure to fluoride can potentially elicit deficiencies in the biologically active forms of these ions, thus necessitating cells to increase the concentrations of available free metal ions. Consistently, we found that NaF treatment upregulated the expression of a large number of genes controlling the uptake of iron (e.g., *entCEBA nfeF*, *exbBD*, *feoABC*, etc.) and magnesium (i.e., *mgtA* and *mgtS*).

Furthermore, many upregulated genes are involved in cell envelope stress (e.g., *pspA otsBA*, *treF*, and *osmY*) and lipid biosynthesis (*pgpC*, *ybhP*, *cslB*, and *ybhN*). Many of them are positively regulated by the nucleotide alarmone ppGpp. It seems likely that their upregulation is a result of the ppGpp-mediated stress response caused by defects in ATP production upon NaF exposure. This notion is well supported by a recent study reporting novel roles for ppGpp in bacterial physiology (35). In contrast, the putative increase in ppGpp level in response to NaF treatment might account for the downregulation of numerous genes involved in translation (e.g., *infA*, *rrf*, *prfC*, etc.).

Further examination of our data revealed that the mentioned transcriptional/posttranscriptional regulators likely contribute to NaF-dependent E. coli gene regulation under anaerobic conditions. In particular, we found that downregulation of the dual regulator H-NS (encoded by *hns*) appears to relieve (activate) expression of several genes/operons (e.g., *proVWX*, *osmC*, and *osmY*) involved in the osmotic stress response, and concomitantly prevent its role as a transcriptional activator, thus reducing transcription (downregulating) of *cydAB* (energy production) and *degP* (periplasmic endoprotease), among others. Similarly, downregulation of Fis, a transcriptional activator of many operons, including those coding for tRNA precursors, is consistent with an observed decrease in level of numerous tRNAs (Table 2; cluster 5).

In addition to transcription factors, a number of global regulators (Rnc, Poly[A] polymerase I and RNA pyrophosphohydrolase) act at the posttranscriptional level. As the level of the corresponding genes (*rnc*, *pcnB* and *rppH*, respectively) is downregulated in the presence of NaF, it can be envisaged that NaF treatment slows down RNA processing and turnover.

Notably, we observed that the abundance of numerous transcripts increased upon NaF treatment. Although transcript abundances can generally increase or decrease in response to various environmental signals, the NaF-mediated reduction in ATP level we observed for E. coli under anaerobic conditions—and the potentially concomitant decrease in ATP-dependent metabolic activities such as translation, transcription, and degradation of biomolecules (e.g., DNA and RNA)—suggest that profoundly increased abundance of certain transcripts might not be due to a considerably higher rate of transcription (which, in fact, should be less efficient upon ATP depletion). Instead, it could be a result of RNA stabilization as part of the posttranscriptional control playing essential role under various stress conditions (36, 37). Indeed, our analysis of the top upregulated transcripts indicated that a large proportion of them contain stable secondary structures (such as REPs elements) that impede 3′ to 5′ end RNA decay by exonucleases. In fact, we found that a large percentage (~40%) of highly stabilized transcripts (70 versus 0 mM NaF) carried REPs (Fig. 3D).

Although RNA degradation by 3′ exonucleases (e.g., PNPase or RNase II) can be promoted by RhlB-mediated RNA unwinding or 3′ end polyadenylation, both those reactions are ATP-dependent and, consequently, they should be less efficient in circumstances where fluoride depletes ATP. Consistently, our Northern blot hybridization using a probe targeting 5′ regions of several upregulated transcripts (i.e., *osmC*, *proP*, *efeO*, and *yghA*) revealed stabilization of these REP-containing transcripts in a dose-dependent manner upon NaF treatment, and their abundances subsequently decreased after dilution of the cultures with fresh medium lacking NaF (Fig. 3Ca–d). Previous studies have revealed that a number of osmotic stress-related genes (i.e., *proP*, *proV*, *otsBA*, *osmC*, *osmY*; Table 2) upregulated by NaF treatment were also upregulated by 0.4 M NaCl at both transcript levels (24, 38). However, in contrast to 0.4 M NaCl, we only used 70 mM NaF, i.e., at a considerably lower concentration (>5-fold). Therefore, it seems more likely that transcript stabilization *per se* (rather than osmotic stress) is responsible for the observed regulation. Moreover, our additional experimental data (Fig. S1) support the conclusion that the increased levels of *osmC*, *proP*, *efeO*, and *yghA* transcripts are mainly due to exposure to fluoride rather than due to increase in osmolarity.

Further analysis on two selected transcripts, i.e., *osmC* (carrying 1 REP) and *yghA* (carrying 5 REPs), revealed that the observed changes in their abundance were likely caused by enhanced RNA stability, as manifested by the prolonged half-lives of the transcripts in the presence of 70 mM NaF (Fig. 4Ba and Bb and 4Fa and Fb). Moreover, the initial increase in transcript level of full-length *osmC* we detected (Fig. 4Bb) is likely attributable to its efficient stabilization in the presence of 70 mM NaF before complete rifampicin-mediated transcriptional inhibition is attained. To date, studies on the structure of RNA polymerase and inhibition by rifampicin have predominantly focused on the holoenzyme containing the most abundant sigma factor 70, which is responsible for recognizing most E. coli genes. However, in E. coli cells, there are at least seven alternative sigma factors responsible for recognizing different types of promoters (39), and it has been reported that transcription from the sigma70- and sigma32-dependent promoters is differentially inhibited by rifampicin (40). Furthermore, it remains unclear if binding of different σ subunits to the core enzyme represents a simple case of factor substitution that has no effect on core structure or if their binding significantly alters the conformation of the core under different growth conditions. We speculate that transcription of *osmC* under anaerobic conditions is dependent on an alternative sigma factor and so it is less sensitive to rifampicin, particularly upon NaF treatment. As a result, the remaining percentage of FL *osmC* mRNA increases within a short time after rifampicin treatment, as shown in Fig. 4Bb.

Our experimental data support the notion that regulation of *osmC* transcript levels is exerted at the posttranscriptional level by reducing the degradation rate of full-length transcript and its decay intermediate (Fig. 4Ba and Bb and 4Fa and Fb).

The main pathway of RNA turnover in E. coli is controlled by the endoribonuclease RNase E, a primary component of the multienzyme ribonucleolytic complex termed the RNA degradosome (16, 17). Other major components of degradosomes include enolase, RhlB RNA helicase and the 3′-to-5′ exonuclease polynucleotide phosphorylase (PNPase) (15–17). RNA degradosome assembly is required for efficient RNA turnover under both aerobic and anaerobic conditions. We have recently elucidated a mechanism by which E. coli uses enolase-bound degradosomes and the small RNA DicF to alternate from rod-shaped to filamentous form in response to anaerobiosis (19). However, despite some advances, our understanding of how RNA degradation is regulated in bacteria under anaerobic conditions remains limited. To explore anaerobic RNA decay further, we examined if RNase E is required to degrade E. coli *osmC* and *yghA* transcripts using an RNase E temperature-sensitive N3431 strain (25) (Fig. 4Ca and Cb and 4Ga and Gb).

As described above, the *osmC* and *yghA* genes carry REPs at the 3′ end. Although REPs possess stable structures that inhibit 3′-to-5′ exonucleolytic degradation of the cognate transcripts, and/or restrict cleavage at potentially susceptible RNase E sites (41), E. coli deploys mechanisms to overcome the stabilizing effect of REPs. For instance, it was reported previously that degradosomes exert ATP-dependent activity that aids in the unwinding of structured RNAs by RhlB helicase, facilitating their subsequent degradation by PNPase (17) in the 3′ to 5′ direction (42). Alternatively, REP-containing RNAs can be destabilized by polyadenylation, which is normally catalyzed by poly(A) polymerase I (PAPI) that adds poly(A) tails to the 3′ end of E. coli transcripts, thereby expediting exonuclease binding and subsequent digestion of the RNAs by PNPase, RNase II, and RNase R (43).

Given that *osmC* and *yghA* transcript abundance increased considerably upon NaF fluoride treatment (Fig. 4Ba and Bb and 4Fa and Fb), it seems likely that this effect could be caused, at least in part, by inhibition of their RhlB-dependent and/or PAPI-dependent decay, presumably due to NaF-dependent ATP depletion. Moreover, degradation of both transcript types may be dependent on RNase E, the key player in E. coli mRNA decay, whose quaternary structure regulates RNA turnover in a substrate length-dependent manner (44). Indeed, our assessment of RNA half-lives in wild-type (N3433) and temperature-sensitive RNase E mutant (N3431) strains revealed enhanced stability of *osmC* and *yghA* transcripts upon inactivation of RNase E (i.e., in N3431 at the nonpermissive temperature). This finding indicates that RNase E-dependent mechanisms play a critical role in degrading structured RNAs under anaerobic conditions. It will be interested to address in future, whether NaF treatment affects these mechanisms by altering RNase E localization or its tertiary structure under anaerobic conditions.

Collectively, our study reveals that ATP depletion under anaerobic conditions upon exposing E. coli to 20 mM or 70 mM NaF leads to dramatic changes in gene expression. Moreover, NaF-induced upregulation of certain genes in E. coli is likely exerted at the posttranscriptional level, apparently by impeding ATP-dependent unwinding (or polyadenylation) of the respective transcripts. Inhibition of these pathways prevents efficient 3′-to-5′ degradation by exonucleases of the structured RNAs. This scenario is supported by the increased half-lives (chemical stabilization) of structured RNAs upon NaF treatment, as demonstrated by Northern blotting analysis of *osmC* and *yghA* transcripts, which are protected from exonucleolytic decay at their 3′ ends by REP sequences. Furthermore, we report that degradation of these transcripts is RNase E-dependent, implying that anaerobic turnover of structured RNAs likely involves the combined action of both exo- and endoribonucleases.

## MATERIALS AND METHODS

### Bacterial strains, growth conditions and sodium fluoride treatment.

E. coli MG1655 K-12 strain was grown anaerobically in a 1 L Winpact bench-top fermentor (Major Science Inc., USA) with M9 medium (45) supplemented with 0.4% glucose, as described previously (19, 46). In brief, the overnight culture was subcultured in a 1 L fermentor vessel containing M9/glucose medium that had initially been incubated for ~12 h with a constant flow rate of 0.5 L/min of sparged pure nitrogen gas to maintain anaerobic conditions. Then, overnight cultures that had been diluted to an optical density at 460 nm (OD_460_) of ~0.05 were added. The pH, temperature, and agitation were maintained at 7.0, 37°C, and 200 rpm, respectively.

For our ATP depletion experiments, 500 mL of each culture was grown anaerobically to OD_460_ ~0.4, and then NaF was added to obtain a final concentration of 20, 40, 50, 60, 70, 80, or 160 mM, whereas the control culture (0 mM NaF) remained untreated. After incubating the cultures for 8 min, aliquots were withdrawn for ATP measurement and microscopy imaging.

It was reported previously that fluoride ions present at millimolar concentrations in bacterial culture media inhibit cell growth in E. coli under aerobic conditions (47). We tested the effect of fluoride on growth of E. coli under anaerobic conditions (Fig. S2; compare 0 mM versus 20 to 160 mM). We found that sodium fluoride likewise inhibits cell growth, leading to its arrest at a concentration of 70 mM NaF. In our experiments, we treated cells for 8 min to ensure that cells are alive and physiologically active.

Two sets of cultures were prepared in M9/glucose medium for tiling microarray (Fig. 1C). The first one (Cd cultures) included untreated cells (0 mM NaF, control) and two cultures treated with 20 or 70 mM NaF, respectively. The second set of cultures underwent partial reversal of the effects of NaF treatment by diluting the Cd cultures with fresh M9/glucose medium and incubating them to reach the cell density of the original (undiluted) Ce cultures (Fig. 1C). To obtain both sets of cultures, 2 mL M9/glucose medium inoculated with a single E. coli colony was incubated overnight at 37°C to obtain the starting culture (Ca) for inoculation into 50 mL M9/glucose medium placed in a 250 mL Erlenmeyer flask and grown overnight under the same growth conditions, resulting in Cb culture. This later was transferred to a 1 L fermentor vessel containing 300 mL of M9/glucose (maintained under anaerobic conditions at an OD_460_ of ~0.05), and the cells were grown anaerobically until the OD_460_ reached ~0.4 (culture Cc), before transferring the fermentor vessel to an Anaerobic System Glove Box to maintain the anaerobic conditions. Aliquots (100 mL each) of culture Cc were transferred to three 500 mL Erlenmeyer flasks (representing the Cd cultures), and NaF was added to two of them to obtain a final concentration of 20 mM or 70 mM, with the third culture remaining untreated (control). After incubating all Cd cultures for 8 min, aliquots were withdrawn for RNA isolation, and the remaining NaF-treated cultures (20 mL) were diluted (1:5) with fresh M9/glucose medium to reduce the NaF concentration to 4 or 14 mM, respectively. These diluted cultures (Ce) were further incubated under the same conditions and, when they reached an OD_460_ of ~0.4, aliquots were withdrawn for RNA isolation. These experiments were performed on three biological replicates of each culture.

To measure RNA half-lives, MG1655 cultures were grown anaerobically as described above. Then each 0 mM and 70 mM NaF (8 min) culture was treated with rifampicin to inhibit new RNA synthesis. In the experiments carried out on the *rne* temperature-sensitive N3431 and isogenic wild-type N3433 strains (25), the cultures were grown anaerobically at 30°C. Then, each culture was treated with rifampicin or shifted to 44°C for 1 h prior to addition of rifampicin (0.5 mg/mL).

### ATP measurement.

ATP was extracted from cells using perchloric acid according to a previously described procedure (48). In brief, before withdrawing an aliquot of culture, 200 μL of ice-cold 3.0 N HClO_4_ was placed into an Eppendorf tube and kept on ice, to which the culture aliquot (800 μL) was added. The tube was vortexed and kept on ice for 10 min, before adding 200 μL of 3.0 N KOH in 0.3 M HEPES (pH 7.8) to neutralize HClO_4_, to stabilize the ATP against acid-catalyzed hydrolysis, and to precipitate KClO_4_. After the precipitate had formed and settled, the supernatant was removed and stored frozen at −20°C for later assays. ATP level was measured by luciferase assay in a luminometer (EnSpire multilabel plate reader, PerkinElmer) using an ATP detection kit (Invitrogen) according to the manufacturer’s instructions. In brief, the assay was carried out on black flat-bottomed 96-well microtiter plates. Each sample (10 μL) and 90 μL of a standard reaction solution were mixed in the individual wells and luminescence (a.u.) was recorded at 570 nm. Changes in ATP level were calculated and the percentage of ATP (normalized to untreated control) was determined. All measurements of ATP level were performed in triplicate.

### Microscopy imaging.

For cell imaging, 2 μL of cell culture was placed on the middle of a glass plate, then covered with a cover slide and sealed. Differential interference contrast (DIC) images were captured using an Axio Imager Z1 (Carl Zeiss) microscope at 100× magnification. The images were analyzed in ZEN Blue Edition software (Carl Zeiss).

### RNA isolation and Northern blot analysis.

Total RNA was extracted by means of the hot acidic phenol method (21). In brief, aliquots (43 mL) of cell cultures grown anaerobically in M9/glucose medium as described above were mixed with cold stop solution (5% phenol in ethanol) at an 8:1 ratio. The cells were collected by centrifugation (4,000 *g*, 4°C, 15 min), suspended in 2 mL KJ medium (50 mM glucose, 25 mM Tris-HCl pH 8.0, 10 mM EDTA pH 8.0, 100 mM NaCl), then added into 2 mL boiling lysis buffer (0.2 M NaCl, 20 mM Tris-HCl pH 7.5, 40 mM EDTA, 0.5% SDS), and incubated in boiling water for 30 sec before adding 2 mL acidic phenol (pH 4.5). The contents of each tube were gently mixed by inverting the tube ~20 times. Total RNA was extracted into aqueous phase by centrifugation (4,000 *g*, 4°C for 1 h). Then, the RNA was precipitated by adding 1 volume of isopropanol and 1/10 volume of 3 M sodium acetate (pH 7.8). The resulting RNA suspensions were stored at −20°C. Prior to use, the RNA was precipitated by centrifugation (30,000 *g*, 4°C for 15 min), washed with 70% ethanol, centrifuged again (30,000 *g*, 4°C for 15 min), and suspended in 20 μL water.

The purified RNA was first analyzed by Bioanalyzer (Agilent 2100). A ratio of rRNAs [23S/16S] > 1.4 was considered to reflect RNA of high quality, so it was used for subsequent tiling array analysis.

For Northern blot analysis, aliquots of total RNA (10 μg) were individually mixed with equal volumes of 2× RNA loading dye (0.03% bromophenol blue, 0.03% xylene cyanol FF, 0.5 mM EDTA in formamide), incubated at 65°C for 10 min, chilled on ice, and separated on 3.5% or 6% polyacrylamide-urea gels. The fractionated RNAs were transferred to ZetaProbe blotting membranes (Bio-Rad) at 400 mA (60 min at 4°C) in 0.5×TBE buffer and UV cross-linked (1,200 mJ) using a Stratalinker UV Crosslinker 2400 system (Stratagene). RNAs were stained with 0.3% methylene blue/0.3 M sodium acetate solution (pH 5.2) for 1 min and further washed with miliQ water for 5 min on a shaker, and then scanned using an Epson Perfection 4990 photo scanner. The membranes were cut into smaller pieces and, after prehybridization with ULTRAhyb hybridization buffer (Ambion) at 65°C for 12 h, they were further hybridized with the specific oligonucleotide probes internally labeled with [α-^32^P] ATP oligodeoxynucleotide or 5′-labeled with [γ-^32^P] ATP (Table S1). The internally labeled RNA probes were synthesized using an *in vitro* transcription kit (MAXIscript, Ambion) and 5′-labeled DNA probes were labeled using T4 polynucleotide kinase (T4 PNK, Thermo Scientific) according to the manufacturer’s instructions. DNA templates for transcription of individual RNA probes were generated by PCR using gene-specific primers (Table S1). Radioactive probes were purified using a MicroSpin G-25 column (GE Healthcare). We used RiboRuler high and low range RNA ladders (Thermo Scientific) to estimate the approximate size of RNAs. After hybridization at 65°C (internally labeled probes) or 42°C (oligonucleotide probes) overnight, the membranes were washed twice with preheated wash buffer (5× SSC with 0.5% [wt/vol] SDS) at 65°C (internally labeled probes) or 42°C (oligonucleotide probes) and exposed to Phoshor imaging plates (FujiFilm) at −80°C. The RNA bands were detected using a Typhoon FLA 9000 biomolecular imager (GE Healthcare), and the relative amount of individual RNA species in each band was calculated by normalizing its signal to 5S or 16S rRNA signals using as controls for our Northern blots. Ribosomal RNAs are very stable and therefore their steady-state intracellular levels are almost the same, which was supported by their expression profiles in our tiling array data. In other words, all three ribosomal RNAs (i.e., 23S, 16S, and 5S) belong to clusters 1 or 2 (Fig. 2), which indicates that their levels were not significantly affected by NaF treatment, so they can be used as loading controls for Northerns. For transcript level comparisons we used the same cell mass.

### Tiling array.

The purified RNA was first analyzed by Bioanalyzer (Agilent 2100), with a ratio of rRNAs (23S/16S) >1.4 being considered of sufficiently high quality for tiling array analysis. The RNA was then converted to labeled cDNA, which was hybridized to a whole-genome tiling array (NimbleGen) using standard NimbleGen operating protocols. In brief, double-stranded cDNA was synthesized using a Nimblegen cDNA synthesis kit from 10 μg total RNA and up to 1 μg of the resulting cDNA was plabeled with Cy3-9mer primers by using a Nimblegen labeling kit. Following labeling, 5 μg of Cy3-cDNA was hybridized for 18 h at 42°C in a MAUIHybridizer unit with a Nimblegen 385K E. coli tiling array by means of the Nimblegen hybridization kit. Afterwards, the array was washed using Nimblegen washing buffers, followed by drying on a microarray dryer. The microarray slides were scanned using the Axon GenePix 4200A microarray scanner at 5 μm resolution and photomultiplier tube (PMT) at 480 V. The scanned images were processed using NimbleScan software. The array images were further processed using Nimblegen's standard protocol for Nimblescan ChIP data extraction.

### Bioinformatics.

The expressions for each gene were quantified by summarizing the tiling-array probe signals (subColSummarize) and normalizing across all the samples (“normalize.quantiles”) using the R package “preprocessCore” (version 1.58.0). For each sample, relative fold changes of each transcript were calculated by comparing it to the gene expression value of the control sample. The k-means clustering analysis (k = 6) was performed by running the R function “kmeans” on the log_2_-transformed median fold changes in expression across all samples. We applied STRING analysis (49) to obtain the interaction network of differentially expressed genes. The Biocyc Database Collection (https://biocyc.org) was used to identify REPs in the E. coli MG1655 K-12 strain.

### Quantification and statistical analysis.

Integrated band intensity was quantitated using Fiji (50) software based on the intensity of signals obtained by scanning the phosphor image plates. Optical density was obtained using a Prema PRO-739 visible spectrophotometer. All statistical tests were performed using GraphPad Prism version 9.0.

### Data availability.

The tiling array data and related information have been deposited to NCBI GEO (accession no. GSE211579).
