# Supplementary material for: Sodium Fluoride Exposure Leads to ATP Depletion and Altered RNA Decay in Escherichia coli under Anaerobic Conditions
Source: Microbiol Spectr. 2023 Mar 20;11(2):e04158-22. doi: 10.1128/spectrum.04158-22 (PMC10100675; doi:10.1128/spectrum.04158-22)
Supplement: Supplemental file 1 — Supplemental material. Download spectrum.04158-22-s0001.pdf, PDF file, 0.5 MB [file spectrum.04158-22-s0001.pdf]

# Figure S1

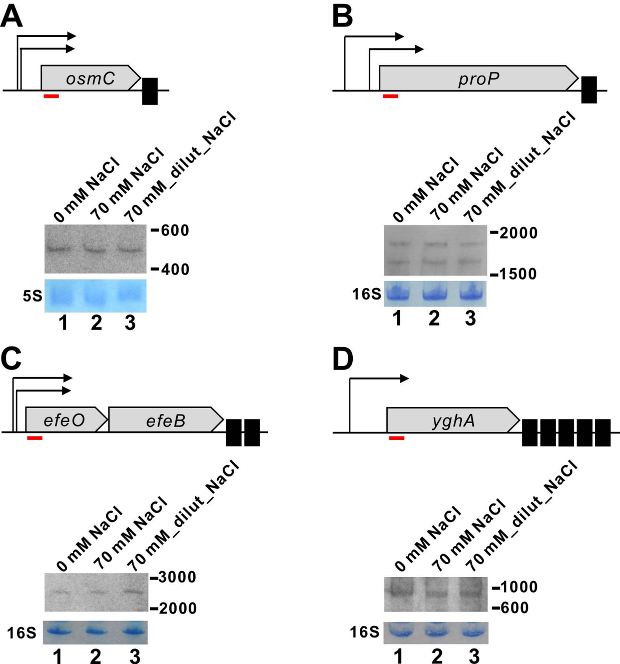
A
osmC
0 mM NaCl
70 mM NaCl
70 mM_dilut_NaCl
600
400
5S
1
2
3
B
proP
0 mM NaCl
70 mM NaCl
70 mM_dilut_NaCl
2000
1500
16S
1
2
3
C
efeO
efeB
0 mM NaCl
70 mM NaCl
70 mM_dilut_NaCl
3000
2000
16S
1
2
3
D
yghA
0 mM NaCl
70 mM NaCl
70 mM_dilut_NaCl
1000
600
16S
1
2
3

## Figure S2

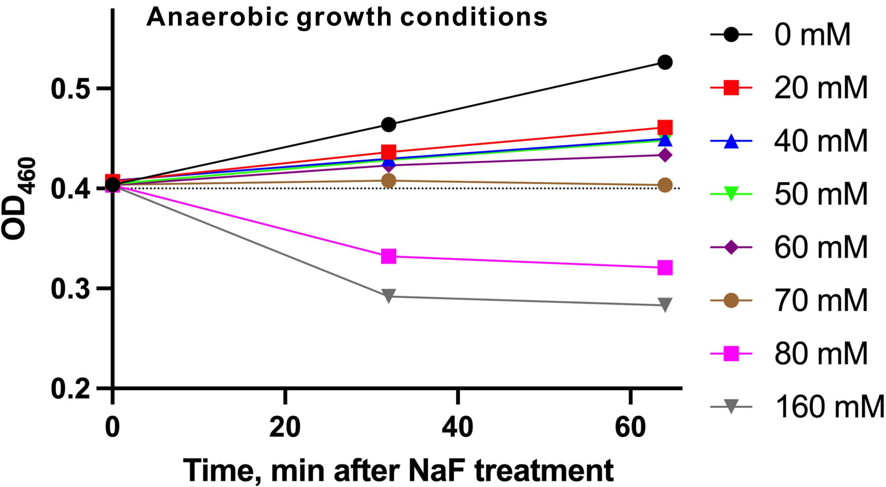

Anaerobic growth conditions
0.5
0.4
0.3
0.2
OD460
0
20
40
60
Time, min after NaF treatment
0 mM
20 mM
40 mM
50 mM
60 mM
70 mM
80 mM
160 mM

# Supplementary figure legends

**Fig. S1. Steady-state levels of *osmC* (A), *proP* (B), *efeO* (C) and *yghA* (D) RNAs upon an increase (0 and 70 mM) and subsequent decrease [14 (70 mM_dilut)] in concentration of NaCl.** Hybridizations were performed with internally-labeled probes. The 5S and 16S rRNAs served as internal loading controls. RiboRuler RNA Ladder (Thermo Scientific) was used as a size marker. Schematic representations of the *osmC*, *proP*, *efeO-efeB* and *yghA* operons are shown on top of each Northern blot. Repetitive extragenic palindromic sequences (REPs) are depicted as black rectangles. The regions complementary to the probes are indicated by thin red lines under the 5'-end of each transcript.

**Fig. S2. Optical densities of *E. coli* cultures grown under anaerobic conditions after NaF treatment [0 (control) to 160 mM].** The dotted black line corresponds to $OD_{460}$ ~ 0.4. Error bars represent the standard deviation of three biological replicates.

**Table S1.** Oligonucleotide primers used to amplify templates for *in vitro* transcription of RNA probes and oligonucleotides used as DNA probes for Northern blot detection used in this study. Related to Figures 3 and 4.

| Name | Sequence (5' to 3') | Product length (bp) | Target |
|---|---|---|---|
| osmC_fw | ACAATCCATAAGAAAGGTCAGGCAC | 170 | MG1655genome |
| osmC_rev | TAATACGACTCACTATAGGGCAGTTCTTCAG GGTTGGTTCC | | |
| proP_fw | CTGAAAAGGAAAAAAGTAAAACCGA | 167 | MG1655 genome |
| proP_rev | TAATACGACTCACTATAGGGTGCGTAAGCAA CAAAACCATA AACAC | | |
| efeO_fw | ACCATTAACTTCCGCCGTAACG | 176 | MG1655 genome |
| efeO_rev | TAATACGACTCACTATAGGGTGTTTTCCCGG CGTTAACCGTAA | | |
| yghA_fw | GACCCGACCACGCAGTATTACACT | 155 | MG1655 genome |
| yghA_rev | TAATACGACTCACTATAGGGACGATCTTTCA GGCGACCGCTA | | |
| osmC_REP | CGCCTGACGCGTCATCCGG | - | MG1655 genome |
| yghA_REP | TGCCTGATGCGCTACGCTCATCAGGCCTA | - | MG1655 genome |

fw = Forward, rev = Reverse,

underlined nucleotides in the reverse primer correspond to T7 promoter.
